# Myocardial Infarction-Induced INSL6 Decrease Contributes to Breast Cancer Progression

**DOI:** 10.1155/2023/8702914

**Published:** 2023-02-07

**Authors:** Yue Zheng, Wenqing Gao, Song Wang, Bingcai Qi, Zhenchang Qi, Xiaomin Hu, Qiang Zhang, Yuheng Lang, Meng Ning, Zhiqiang Luo, Tong Li

**Affiliations:** ^1^School of Medicine, Nankai University, Tianjin 300071, China; ^2^Department of Heart Center, The Third Central Hospital of Tianjin, 83 Jintang Road, Hedong District, Tianjin 300170, China; ^3^Nankai University Affiliated Third Center Hospital, No. 83, Jintang Road, Hedong District, Tianjin 300170, China; ^4^Tianjin Key Laboratory of Extracorporeal Life Support for Critical Diseases, Tianjin, China; ^5^Artificial Cell Engineering Technology Research Center, Tianjin, China; ^6^The Third Central Clinical College of Tianjin Medical University, Tianjin 300170, China

## Abstract

Myocardial infarction (MI) induces early-stage breast cancer progression and increases breast cancer patients' mortality and morbidity. Insulin-like peptide 6 (INSL6) overexpression can impede cardiotoxin-induced injury through myofiber regeneration, playing a significant role in MI progression. To investigate the diverse significance of INSL6 in a variety of malignant tumors, we explored INSL6 through MI GEO dataset and multiple omics data integrative analysis, such as gene expression level, enriched pathway analysis, protein-protein interaction (PPI) analysis, and immune subtypes as well as diagnostic value and prognostic value in pancancer. INSL6 expression was downregulated in the MI group, and overall survival analysis demonstrated that INSL6 could be the prognostic biomarkers in the overall survival of breast cancer (BRCA). INSL6 expression differs significantly not only in most cancers but also in different molecular and immune subtypes of cancers. INSL6 might be a potential diagnostic and prognostic biomarker of cancers due to the high accuracy in diagnostic and prognostic value. Furthermore, we focused on BRCA and further investigated INSL6 from the perspective of the correlations with clinical characteristics, prognosis in different clinical subgroups, coexpression genes, and differentially expressed genes (DEGs) and PPI analysis. Overall survival and disease-specific survival analysis of subgroups in BRCA demonstrated that lower INSL6 expression had a worse prognosis. Therefore, INSL6 aberrant expression is associated with the progression and immune cell infiltration of the tumor, especially in KIRP and BRCA. Therefore, INSL6 may serve as a potential prognostic biomarker and the crosstalk between MI and tumor progression.

## 1. Introduction

Insulin-like peptide 6 (INSL6), as a member of the insulin/relaxin superfamily, is secreted from the endoplasmic reticulum and Golgi in cells ([1], p. 5611; [2], p. 990). INSL6 overexpression can impede cardiotoxin-induced injury through myofiber regeneration in skeletal muscle-specific Insl6 transgenic mice ([3], p. 36060). Maruyama et al. also reported that continuous INSL6 infusion can reduce isoproterenol-induced left ventricular systolic dysfunction and cardiac fibrosis by regulating liver X receptor/retinoid X receptor signaling ([4], p. e008441), suggesting that INSL6 may be a potential biomarker in cardiovascular diseases, such as myocardial infarction.

Currently, coronary artery disease (CAD) is still one of the leading causes of death in patients and contributes to about one in every seven deaths in low- and middle-income countries ([5], p.1151). Acute myocardial infarction (MI) mortality has increased 5.6-fold in the past 30 years, and obesity has become the major cause of morbidity and mortality in patients with some chronic diseases, for instance, diabetes and CAD ([6], p. 229). Conservatively estimated, nowadays, 330 million people develop heart diseases in China and an unacceptable burden of recurrent cardiovascular events needs to be solved. Aseptic inflammation can promote neutrophil extracellular traps (NETs) abundant in the liver, thus increasing metastases in patients with breast and colon cancers ([7], p. 113). Koelwyn et al. reported that MI can epigenetically reprogram Ly6C^high^ monocytes in the bone marrow reservoir to an immunosuppressive phenotype and such monocytes were increasingly recruited to tumors, promoting MI-induced early-stage breast cancer progression and increasing breast cancer patients' mortality and morbidity ([8], p. 1452). Therefore, the crosstalk between MI and tumor progression should be investigated, which may be potential biomarkers to impede tumor progression, thus reducing mortality and morbidity. However, the researches on such crosstalk are little.

In this study, the differentially expressed genes (DEGs) were investigated in MI GEO datasets. Venn diagram was used to obtain the crosstalk between first acute myocardial infarction (FAMI) A, FAMI B, and breast invasive carcinoma (BRCA) and cox regression analysis was used to evaluate the association between the screened DEG expression and BRCA overall survival using Xiantao website. Compared to control, INSL6 expression was downregulated in FAMI A and FAMI B groups, which may impede the inhibitions on tumor progression. Therefore, we examined the INSL6 expression and the diagnostic and prognostic value in pancancer. DEGs between INSL6 high- and low-expression groups in BRCA were also explored to validate whether INSL6 can be the crosstalk between MI and BRCA.

## 2. Methods

### 2.1. Microarray Data and Data Processing

**Figure 1:**
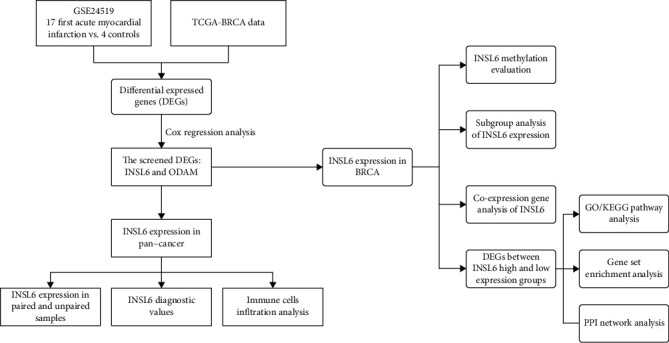
Workflow of processing the datasets.

Using the keywords “myocardial infarction” in “Homo sapiens,” GSE24519 from the Gene Expression Omnibus (GEO) database was investigated, processed with log2 transformation for normalization and analyzed using GEO2R ([9], p. 546). There were 17 patients affected by their very first acute myocardial infarction (FAMI), without any sign of previous cardiovascular sufferance, and 4 controls in GSE24519. Platelets from patient with acute MI within 6 hours of the onset of symptoms were collected, and the blood samples at two time points were named FAMI A and FAMI B. The RNA sequencing was based on the GE Healthcare/Amersham Biosciences CodeLink Human Whole Genome Bioarray, The Cancer Genome Atlas (TCGA) database, and the Genotype-Tissue Expression (GTEx) database by UCSC XENA. The BRCA and other cancer data were all from TCGA (https://portal.gdc.cancer.gov/). The data were downloaded and analyzed using Xiantao website tool (http://www.xiantao.love). A log2|fold change (FC)| > 1 and a *P* value < 0.05 were regarded as the cut-off criteria. The workflow of processing the datasets is shown in Figure 1.

### 2.2. Cox Regression Analysis and Kaplan-Meier Survival Analysis

Using Venn diagram, DEGs were obtained between FAMI A, FAMI B, and BRCA and cox regression analysis was used to evaluate the association between the screened DEG expression and BRCA overall survival using Xiantao website. A *P* value < 0.05 was regarded as the cut-off criteria.

### 2.3. Pancancer Analysis of INSL6 Expression

The gene expression data and RNA sequencing of TCGA pancancer, including paired samples and unpaired samples, were extracted, and the whole data were filtered to remove missing and duplicated results and transformed by log2(TPM + 1) using the Xiantao website tool. A *P* value < 0.05 was regarded as the cut-off criteria.

### 2.4. INSL6-Interacted Proteins and GO/KEGG Pathway Analysis

To investigate INSL6 and its protein interactions, STRING database (https://string-db.org) was used with a combined score > 0.4 ([10], p. D607). The nodes were analyzed using Cytoscape v.3.7.1 ([11], p. 2498). GO and KEGG pathway analyses were also applied to investigate the functions of INSL6-interacted proteins using clusterProfiler in R ([12], p. 25; [13], p. 27; [14], p. 15545; [15], p. 284). A *P* value < 0.05 was regarded as the cut-off criteria.

### 2.5. INSL6 Diagnostic Value Analysis in Pancancer

Receiver operation characteristic (ROC) curve analysis was conducted to investigate the diagnostic performance of INSL6 expression in pancancer, and the area under the curve (AUC) was determined using “pROC” package.

### 2.6. INSL6 Expression Association with Immune Cells

To investigate the relationship between INSL6 expression and immune cells, ssGSEA (GSVA package in R) was used ([14], p. 15545; [15], p. 284), which can provide a critical assessment and integration of 24 immune cells for RNA sequencing samples from TCGA.

### 2.7. INSL6 Methylation Evaluation

INSL6 methylation in KIRP and BRCA was evaluated to investigate the association between INSL6 and methylation site. The data was analyzed from TCGA database and Illumina human methylation 450 databases.

### 2.8. Subgroup Analysis of INSL6 Expression in TCGA-BRCA

To validate the potential effects of INSL6 expressions on BRCA progression, the INSL6 expressions in subgroups were determined and overall survival analysis of subgroups was also carried out. The RNA-seq data and related clinical data in level 3 HTSeq-fragments per kilobase per million (FPKM) format were downloaded from TCGA database, converted to transcripts per million (TPM) read format, and then analyzed after log2 transformation. A *P* value < 0.05 was regarded as the cut-off criteria.

### 2.9. Coexpression Gene Analysis of INSL6 in BRCA

Top 50 coexpression genes positively and negatively related to INSL6 in BRCA were explored. GO/KEGG pathway analysis was used to investigate the enriched pathways of the top coexpression genes. A *P* value < 0.05 was regarded as the cut-off criteria.

### 2.10. DEGs between INSL6 High- and Low-Expression Groups in BRCA

The DEGs between different INSL6 expression groups (low-expression group: 0–50%; high-expression group: 50–100%) in BRCA were analyzed using the deseq2 package. Utilizing Limma, a log2|FC| > 1 and a *P* value < 0.05 were applied as the cut-off criteria. Then, GO/KEGG pathway analyses, as well as gene set enrichment analysis (GSEA), were applied utilizing the “clusterProfiler” package in R. PPI network analysis was used to obtain the hub genes utilizing the Cytoscape plug-in (MCODE and MCC).

## 3. Results

### 3.1. The Same Transcripts between AMI and BRCA

**Figure 2:**
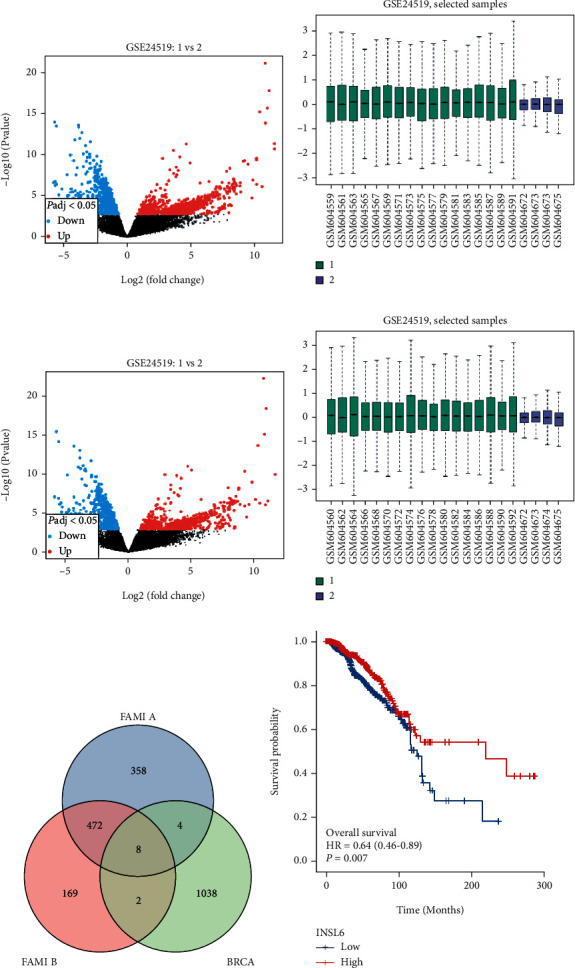
Identification of DEGs between GSE24519 and TCGA-BRCA. (a, b) The volcano plot (a) and bar plot (b) of FAMI A in GSE24519 after log2 transformation for normalization. (c, d) The volcano plot (c) and bar plot (d) of FAMI B in GSE24519 after log2 transformation for normalization. (e) The Venn diagram between FAMI A, FAMI B, and BRCA. (f) The overall survival analysis of different INSL6 expression in BRCA. FAMI: first acute myocardial infarction.

After log2 transformation for normalization, there were 842 DEGs in FAMI A and 651 DEGs in FAMI B in GSE24519 (Figures 2(a)–2(d)). Using Venn diagram, DEGs were obtained between FAMI A, FAMI B, and TCGA-BRCA. There were 14 DEGs with the same transcripts between AMI and BRCA, and 8 DEGs have the same transcripts between FAMI A, FAMI B, and BRCA (Figure 2(e); Table S1).

To investigate which DEG can be a prognostic biomarker, overall survival analysis was utilized and INSL6 (hazard ratio (HR) = 0.64, *P* = 0.007) and ODAM (HR = 0.70, *P* = 0.031) could be the prognostic biomarkers in overall survival BRCA (Figure 2(f); Figure S1). Compared to control, INSL6 expression was downregulated in the FAMI A and FAMI B group, which may impede the inhibitions on tumor progression.

### 3.2. INSL6 Expression in Pancancer

**Figure 3:**
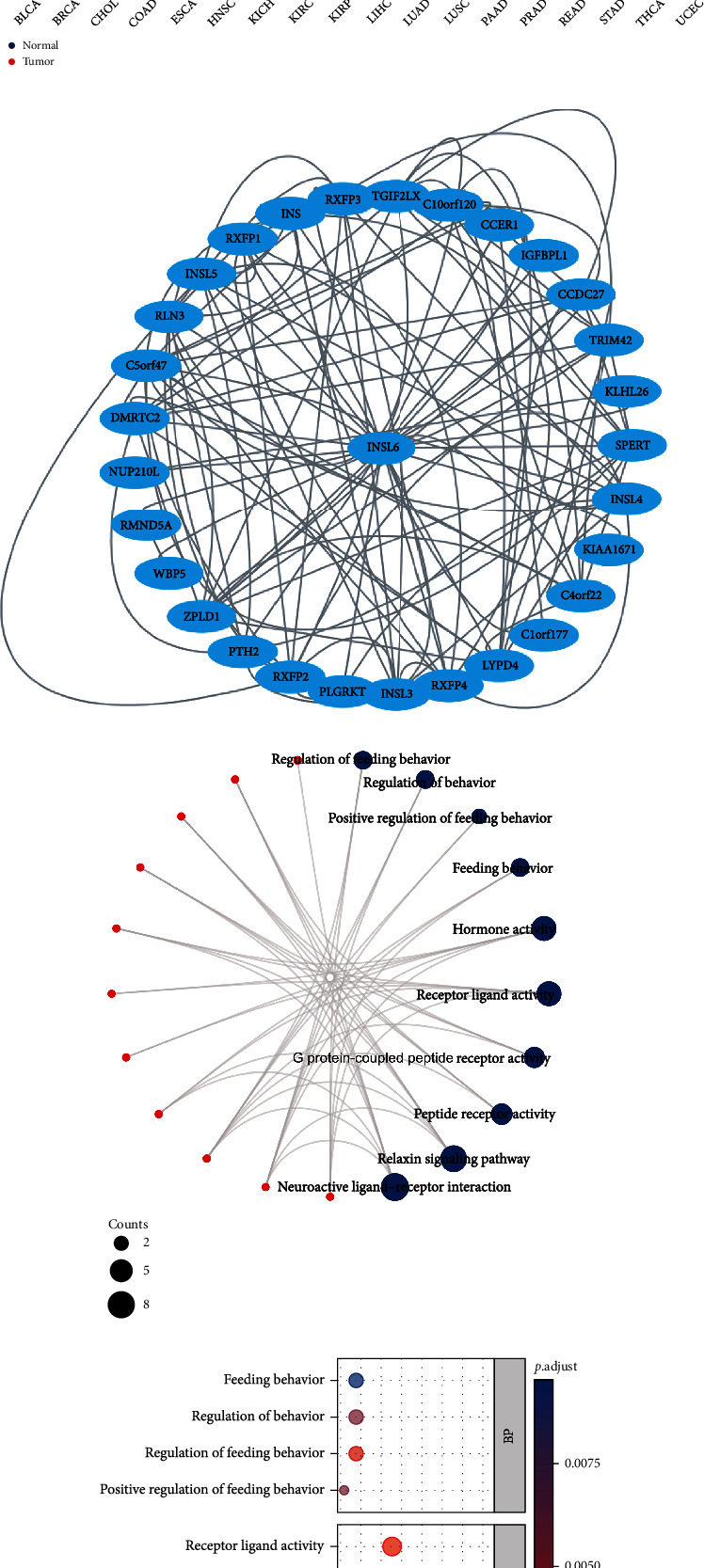
INSL6 expression in pancancer. (a) Unpaired samples in pancancer demonstrated that there were significant differences of INSL6 expression in 20 cell lines in TCGA. (b) Paired samples in pancancer demonstrated there were significant differences of INSL6 expression in 5 cell lines in TCGA. (c) The PPI network of the 50 INSL6-targeting binding proteins. (d, e) The network (d) and bubble diagram (e) of GO/KEGG pathways enriched by the 50 targeting binding proteins.

The gene expression data and RNA sequencing of TCGA pancancer, including paired samples and unpaired samples, were extracted, and the whole data were filtered to remove missing and duplicated results and transformed by log2(TPM + 1) using the Xiantao website tool. Unpaired samples in TCGA demonstrated that INSL6 was highly expressed in BRCA, colon adenocarcinoma (COAD), lymphoid neoplasm diffuse large B-cell lymphoma (DLBC), esophageal carcinoma (ESCA), kidney renal clear cell carcinoma (KIRC), kidney renal papillary cell carcinoma (KIRP), acute myeloid leukemia (LAML), lung adenocarcinoma (LUAD), lung squamous cell carcinoma (LUSC), ovarian serous cystadenocarcinoma (OV), pheochromocytoma and paraganglioma (PCPG), rectum adenocarcinoma (READ), skin cutaneous melanoma (SKCM), stomach adenocarcinoma (STAD), and thyroid carcinoma (THCA), while INSL6 was lowly expressed in adrenocortical carcinoma (ACC), glioblastoma multiforme (GBM), kidney chromophobe (KICH), and testicular germ cell tumors (TGCT) (Figure 3(a)). In addition, paired samples in TCGA demonstrated that INSL6 was highly expressed in BRCA and liver hepatocellular carcinoma (LIHC), while INSL6 was lowly expressed in KICH, KIRC, and THCA (Figure 3(b)).

### 3.3. PPI Network and GO/KEGG Enrichment Analysis

To investigate INSL6 and its protein interactions, the nodes with a combined score > 0.4 were analyzed using STRING and Cytoscape (Figure 3(c)). The list of 50 targeting binding proteins was uploaded into the Xiantao webpage for functional analysis, which were involved in hormone activity, receptor ligand activity, relaxin signaling pathway, and neuroactive ligand-receptor interaction (Figures 3(d) and 3(e); Table 1).

### 3.4. Diagnostic Value of INSL6 in Pancancer

**Figure 4:**
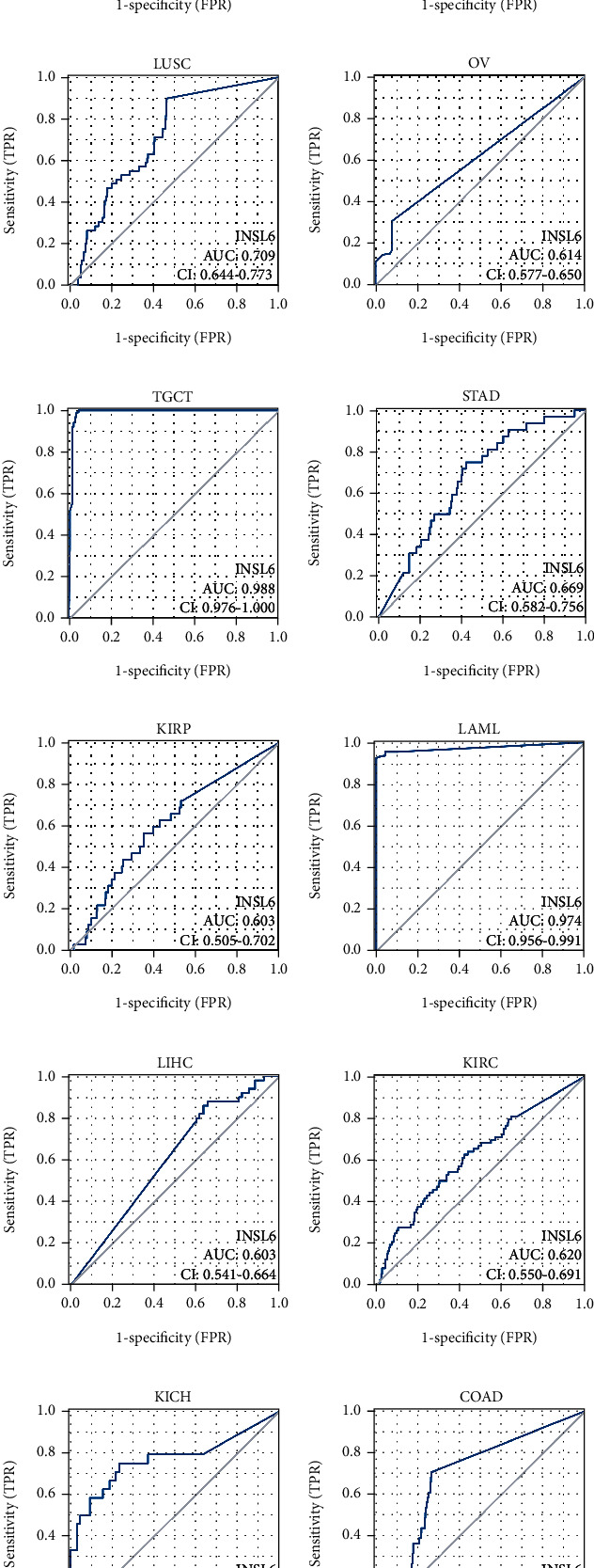
Diagnostic value of INSL6 in pancancer. INSL6 expression can predict 12 cancer types, including BRCA (a), ESCA (b), LUSC (c), OV (d), TGCT (e), STAD (f), KIRP (g), LAML (h), LIHC (i), KIRC (j), KICH (k), and COAD (l).

The ROC was utilized to investigate the diagnostic value of INSL6 in pancancer, which demonstrated that INSL6 expression can predict 12 cancer types (area under the curve (AUC) > 0.6), including BRCA, ESCA, LUSC, OV, TGCT, STAD, KIRP, LAML, LIHC, KIRC, KICH, and COAD (Figure 4; Figure S2). Interestingly, the AUCs of INSL6 expression in TGCT and LAML were 0.988 and 0.974, respectively.

### 3.5. Prognostic Value of INSL6 in Pancancer

**Figure 5:**
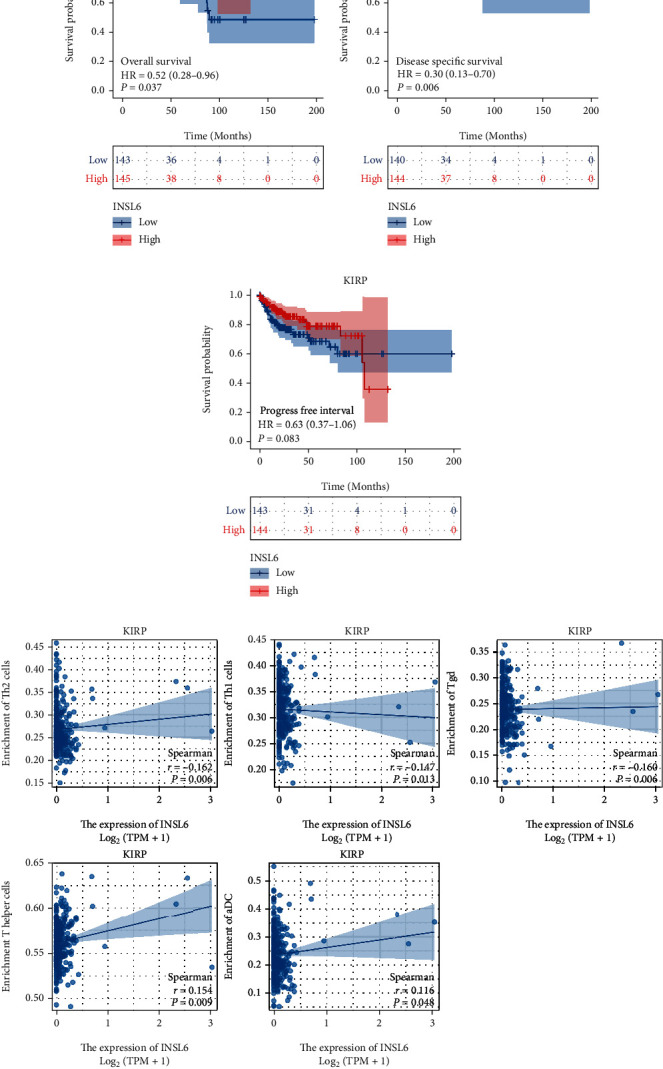
Prognostic value of INSL6 in KIRP. (a–c) The overall survival analysis (a), disease-specific survival analysis (b) and progress-free interval analysis (c) of INSL6 expression in KIRP. (d) The INSL6 expression in KIRP was associated with 5 immune cells, such as Th1 cells, Th2 cells, Tgd, T helper cells, and aDc.

**Figure 6:**
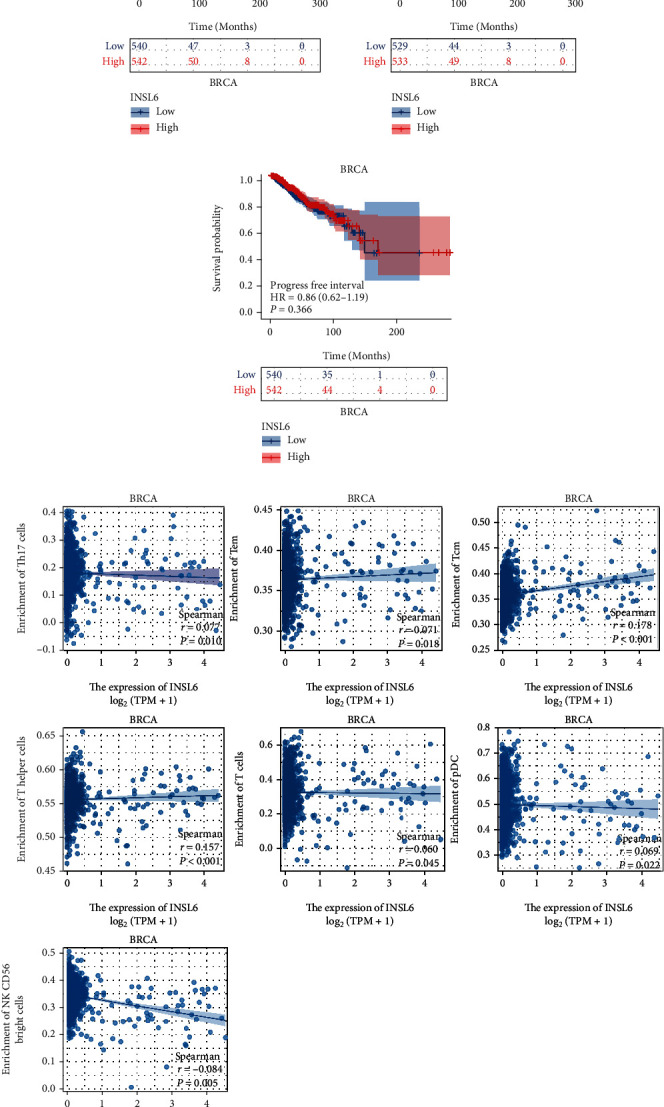
Prognostic value of INSL6 in BRCA. (a–c) The overall survival analysis (a), disease-specific survival analysis (b) and progress-free interval analysis (c) of INSL6 expression in BRCA. (d) The INSL6 expression in BRCA was associated with 7 immune cells, such as T helper cells, Tcm, and CD56bright cells.

The INSL6 expression was notably correlated with the OS and DSS of KIRP and OS of BRCA (Figures 5(a)–5(c) and 6(a)–6(c); Figures S3, S4). Cox regression analysis demonstrated that the lower INSL6 expression had a worse prognosis in KIRP, including OS (HR = 0.52) and DSS (HR = 0.30). Furthermore, the INSL6 expression in KIRP was associated with 5 immune cells, such as Th1 cells, Th2 cells, Tgd, T helper cells, and aDc (Figure 5(d); Figure S5). Cox regression analysis showed that the lower INSL6 expression also had a worse prognosis in OS of BRCA (HR = 0.64). In addition, the INSL6 expression in BRCA was associated with 7 immune cells, such as T helper cells, Tcm, and CD56bright cells (Figure 6(d); Figure S6).

### 3.6. INSL6 Methylation Evaluation in KIRP and BRCA

**Figure 7:**
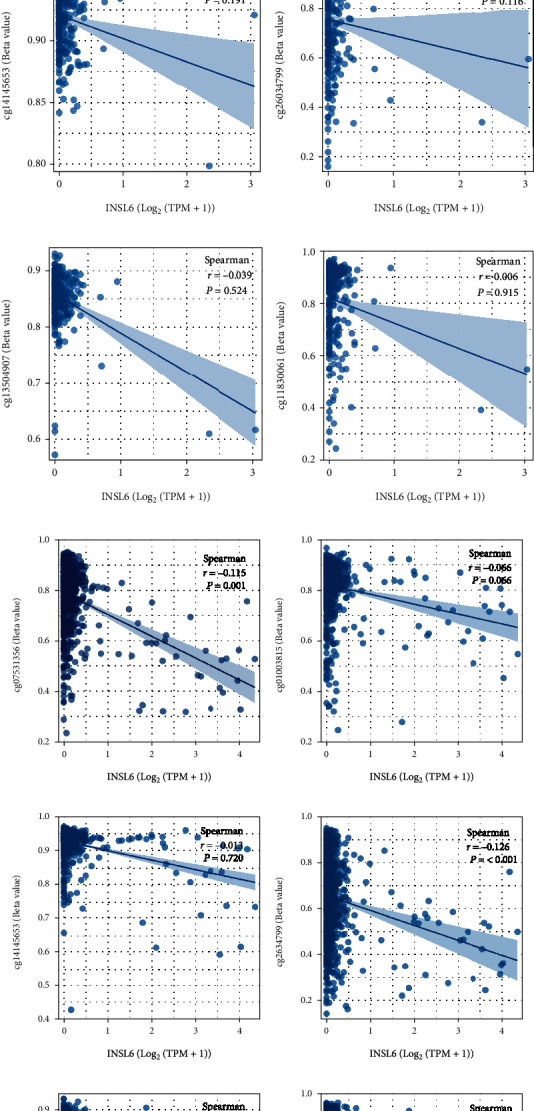
INSL6 methylation evaluation in KIRP and BRCA. (a–f) The association between INSL6 expression and methylation sites in KIRP, including cg07531356 (a), cg01003815 (b), cg14145653 (c), cg26034799 (d), cg13504907 (e), and cg11830061 (f). INSL6 expression was correlated to nothing about methylation in KIRP. (g–l) The association between INSL6 expression and methylation sites in BRCA, including cg07531356 (g), cg01003815 (h), cg14145653 (i), cg26034799 (j), cg13504907 (k), and cg11830061 (l). INSL6 expression was correlated to cg07531356, cg26034799, cg13504907, and cg11830061 in BRCA.

INSL6 methylation in KIRP and BRCA was evaluated to investigate the association between INSL6 and methylation site, which demonstrated that INSL6 expression was correlated to cg07531356, cg26034799, cg13504907, and cg11830061 in BRCA, while INSL6 expression was correlated to nothing in KIRP (Figure 7).

### 3.7. Subgroup Analysis of INSL6 Expression in BRCA

**Figure 8:**
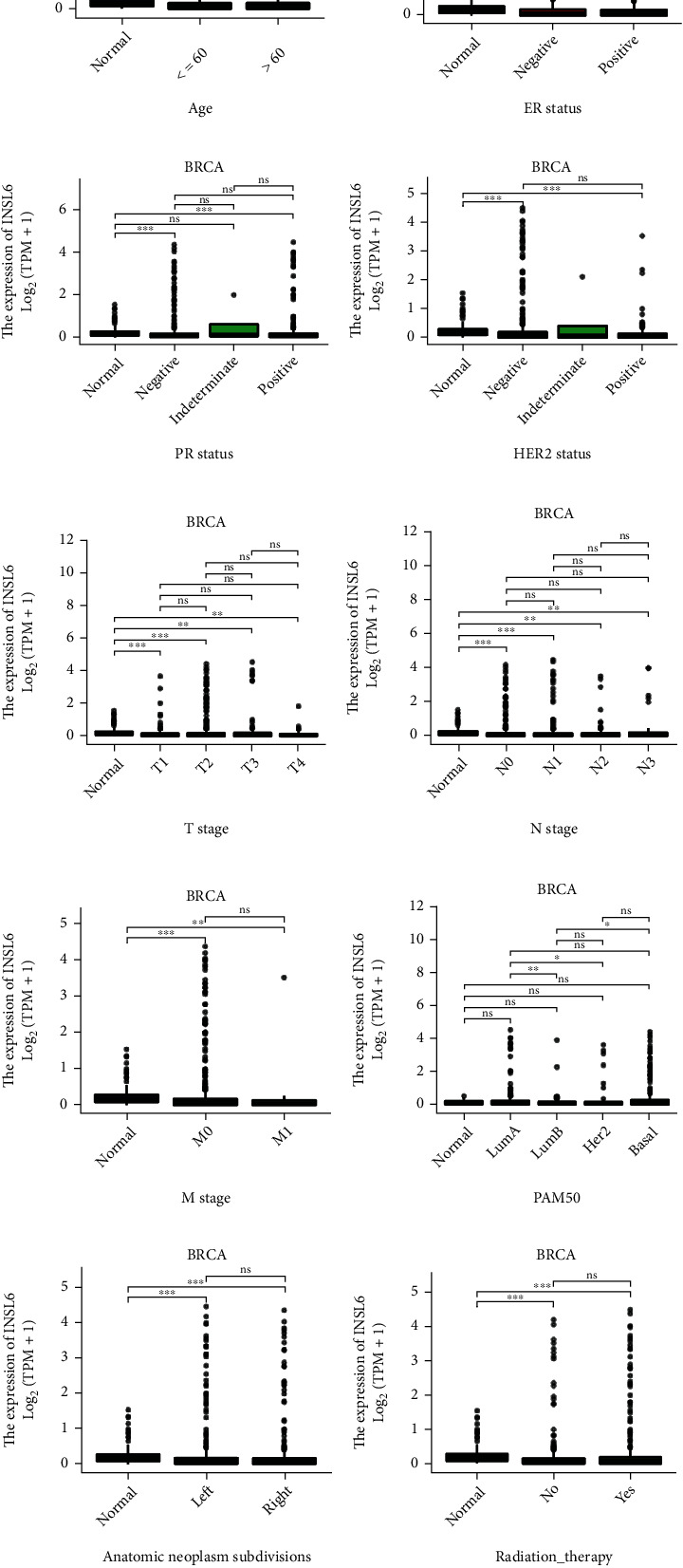
The baseline characters of INSL6 expression in BRCA. The INSL6 expressions in subgroups were explored, including tumor or normal (a), overall survival event (b), disease-specific survival event (c), progress-free interval event (d), age (e), ER status (f), PR status (g), HER2 status (h), T stage (i), N stage (j), M stage (k), PAM50 (l), anatomic neoplasm subdivisions (m), radiation therapy (n), menopause status (o), pathologic stage (p), histologic type (q), and race (r).

**Figure 9:**
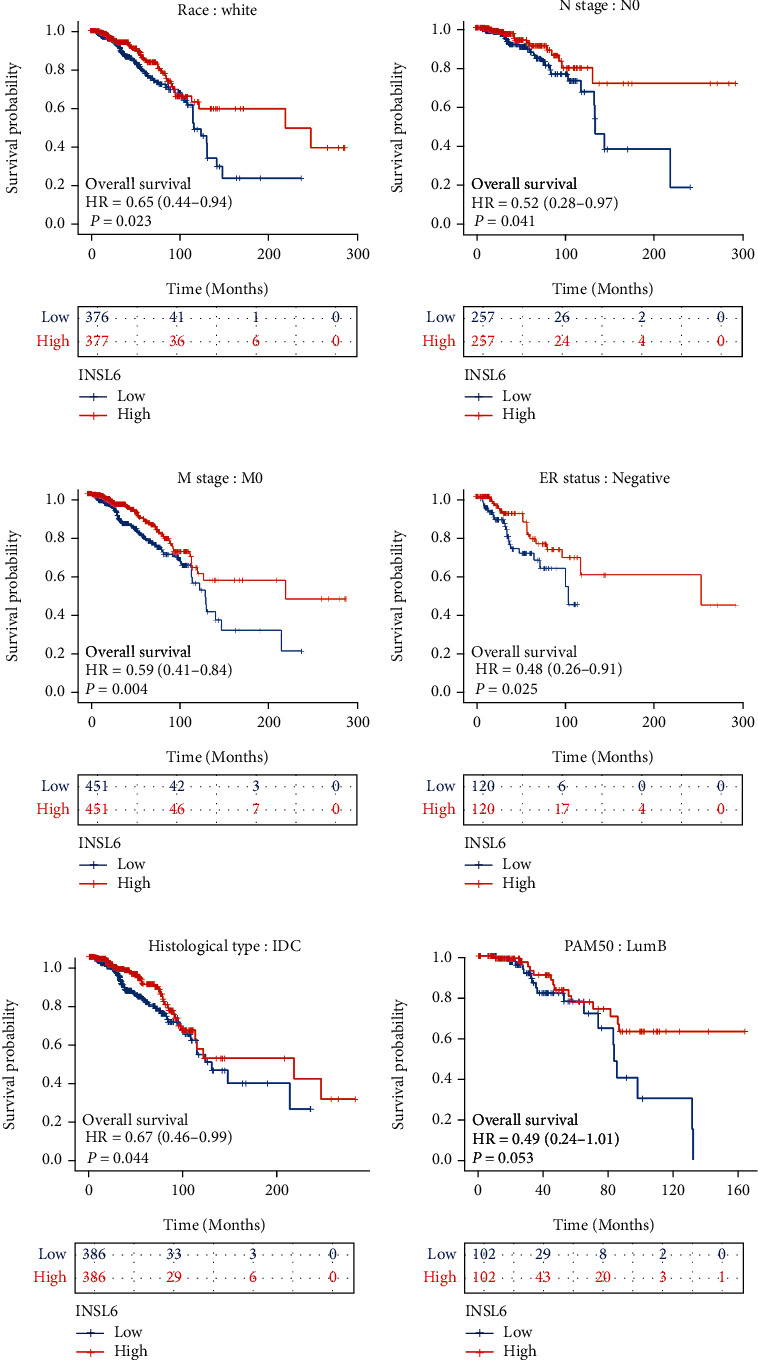
The associations between INSL6 expression and the overall survival in different clinical subgroups of BRCA. (a) Race: white; (b) N stage: N0; (c) M stage: M0; (d) ER status: negative; (e) histological type: IDC; and (F) PAM50: LumB. IDC: infiltrating ductal carcinoma.

To validate the potential effects of INSL6 expressions on BRCA progression, the INSL6 expressions in subgroups were determined (Figure 8; Table S2). After log2 transformation There were significant differences of INSL6 expression in TCGA-BRCA patients' baseline characters, such as age, histological type, and menopause status. Overall survival analysis of subgroups in BRCA was also carried out, which demonstrated that lower INSL6 expression had a worse prognosis in white patients as well as patients with N0, M0, negative ER status, infiltrating ductal carcinoma, or LumB (Figure 9).

Disease-specific survival analysis of subgroups in BRCA demonstrated that lower INSL6 expression had a worse prognosis in patients with M0 or Her2 positive (Figure S7).

### 3.8. Coexpression Gene Analysis of INSL6 in BRCA

**Figure 10:**
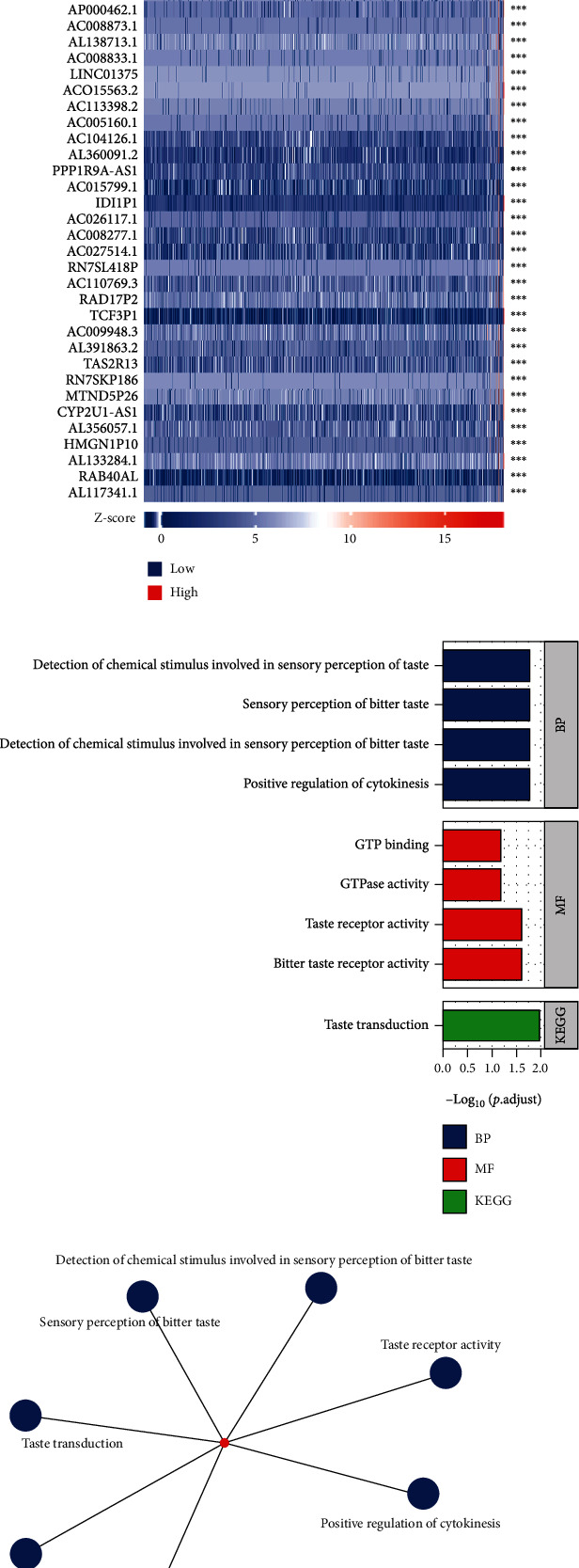
Top 50 genes positively correlated with INSL6 expression in BRCA. (a) The gene coexpression heat map of the top 50 genes positively correlated with INSL6 in BRCA. (b, c) The bar plot (b) and network (c) of GO/KEGG pathways enriched by the top 50 genes positively correlated with INSL6.

**Figure 11:**
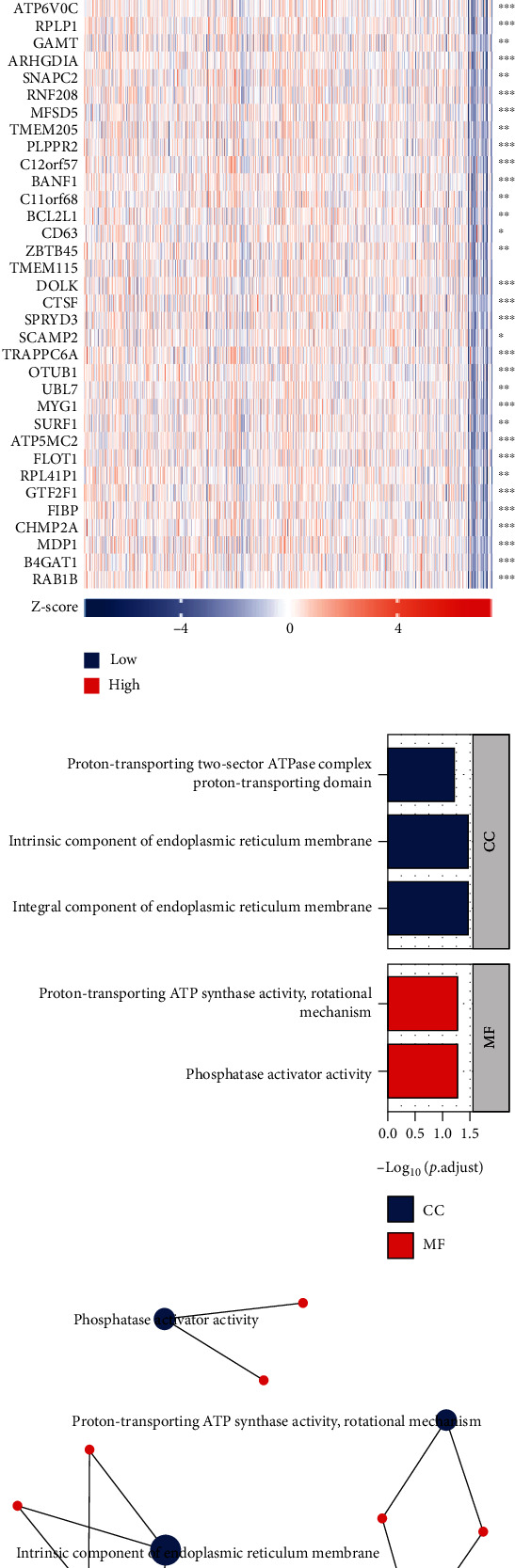
Top 50 genes negatively correlated with INSL6 expression in BRCA. (a) The gene coexpression heat map of the top 50 genes negatively correlated with INSL6 in BRCA. (b, c) The bar plot (b) and network (c) of GO/KEGG pathways enriched by the top 50 genes negatively correlated with INSL6.

Top 50 coexpression genes positively related to INSL6 in BRCA were explored, which were mainly involved in detection of chemical stimulus involved in sensory perception of bitter taste, GTPase activity, and purine ribonucleoside binding (Figure 10; Table 2). Top 50 coexpression genes negatively related to INSL6 in BRCA were also investigated, which were mainly involved in integral component of endoplasmic reticulum membrane, intrinsic component of endoplasmic reticulum membrane, proton-transporting ATP synthase activity, rotational mechanism, and phosphatase activator activity (Figure 11; Table 3).

### 3.9. DEGs between INSL6 High- and Low-Expression Groups in BRCA

**Figure 12:**
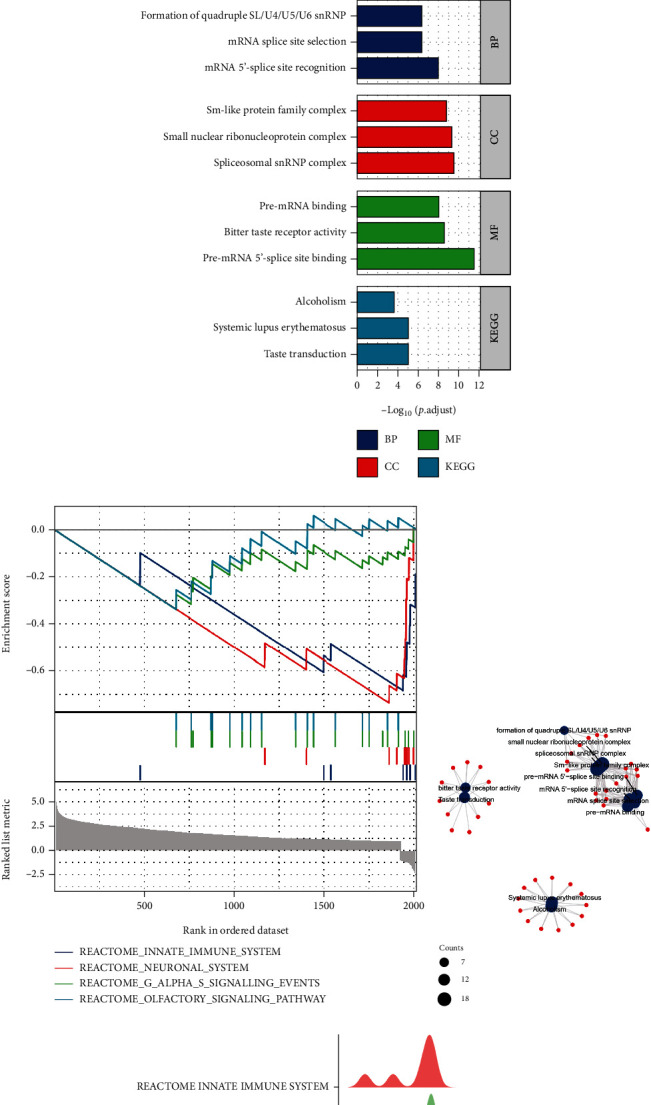
DEGs between INSL6 high-expression and low-expression groups in BRCA. (a) The volcano plot of DEGs between INSL6 high-expression and low-expression groups in BRCA. (b) The bar plot of GO/KEGG pathways enriched by the DEGs. (c) The GSEA of the DEGs between INSL6 high-expression and low-expression groups in BRCA. (d) The network of GO/KEGG pathways enriched by the DEGs. (e) The GSEA mountain figure of the DEGs. GSEA: gene set enrichment analysis.

Using the deseq2 package, 3833 upregulated DEGs and 105 downregulated DEGs were obtained and further GO/KEGG pathway analysis was applied, which were mainly involved in mRNA 5′-splice site recognition, mRNA splice site selection, DNA packaging complex, pre-mRNA binding, and RNA transport (Figures 12(a), 12(b), and 12(d); Table 4). GSEA of DEGs between INSL6 high-and low-expression groups in BRCA was explored, which were mainly enriched in REACTOME_NEURONAL_SYSTEM, REACTOME_G_ALPHA_S_SIGNALLING_EVENTS, REACTOME_OLFACTORY_SIGNALING_PATHWAY, and REACTOME_INNATE_IMMUNE_SYSTEM (Figures 12(c) and 12(e); Table 5).

**Figure 13:**
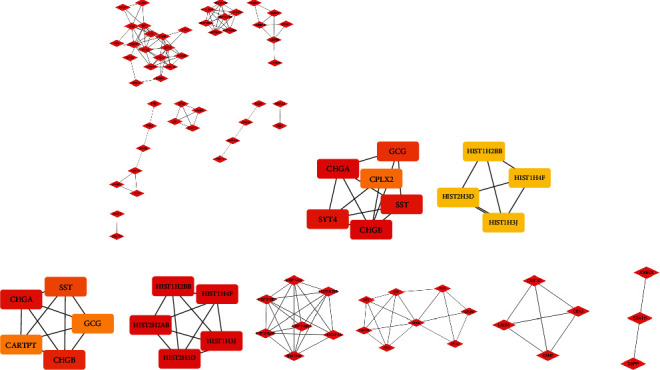
PPI network and the hub genes of the DEGs between INSL6 high-expression and low-expression groups in BRCA. (a) The PPI network of the DEGs between INSL6 high-expression and low-expression groups in BRCA. (b) Top 10 genes screened by the interaction of degrees. (c) Top 10 genes screened by MCC. (d) Four modules of the DEGs analyzed by Cytoscape plugin MCODE.

Furthermore, PPI network analysis was used to obtain the hub genes utilizing the Cytoscape plug-in (MCODE and MCC). There were 4 modules in the network, including CHGB, SST, HIST1H2BB, CSN2, and DSPP (Figure 13).

## 4. Discussion

INSL6 is a member of the insulin gene family, which is containing the insulin family B-chain cysteine motif ([16], p. 1593). Human and rat INSL6 encoded polypeptides of 213 and 188 amino acids, respectively. Besides, human INSL6 was 43% identical to human relaxin H2 in the B- and A-chain regions, which also play a role in muscle biological functions as a relaxin-like peptide ([2], p. 990; [17], p. 14). In this study, the same transcripts between MI and BRCA were investigated and cox regression analysis was used to evaluate the association between the screened DEG expression and BRCA overall survival. Hypothetically, INSL6 expression was downregulated in the FAMI A and FAMI B group, which may impede the inhibitions on tumor progression. Therefore, we examined the INSL6 expression and the diagnostic and prognostic value in pancancer, especially in BRCA. DEGs between INSL6 high- and low-expression groups in BRCA were also explored to validate whether INSL6 can be the crosstalk between MI and BRCA.

INSL6 was firstly investigated in spermatogenesis and reproduction ([18], p. 530; [19], p. 4348; [20], p. 1153). The mass spectrometry analysis also demonstrated that INSL6 can be a novel CUL4B substrate in male germ cells through direct polyubiquitin and degradation by CUL4B E3 ligase ([21], p. 6923). R171H missense mutation of INSL6 could lead to a patient with spermatogenic failure ([22], e455).

The protein INSL6 and its protein interactions were enriched in hormone activity ([20], p. 1153), receptor ligand activity, relaxin signaling pathway ([4], p. e008441), G protein-coupled peptide receptor activity, and neuroactive ligand-receptor interaction ([23], p. 402). The variants of gene combinations about Jak2 and INSL6 may also contribute to the functions of the enriched pathways above mentioned ([24], p. 1; [25], p. 1344).

The ROC analysis demonstrated that INSL6 could predict 12 cancer types, including BRCA, ESCA, LUSC, OV, TGCT, STAD, KIRP, LAML, LIHC, KIRC, KICH, and COAD. Interestingly, the AUCs of INSL6 expression in TGCT and LAML were 0.988 and 0.974, respectively. According to Ji et al., the nomogram with higher prognostic value genes (SEMA6B, SEMA3G, OBP2B, INSL6, and RETN) could predict the 1-year PFS, 3-year PFS, and 5-year PFS of TCGT patients ([26], p. 866).

The INSL6 expression was notably correlated with the OS and DSS of KIRP and OS of BRCA. The INSL6 expression in KIRP was associated with 5 immune cells, such as Th1 cells, Th2 cells, Tgd, T helper cells, and aDc. Cox regression analysis showed that the lower INSL6 expression also had a worse prognosis in OS of BRCA and the INSL6 expression in BRCA was associated with 7 immune cells, such as T helper cells, Tcm, and CD56bright cells. INSL6 deficiency in mice results in a worse myositis phenotype through the elevated infiltration of CD4 and CD8+ T cells and the expression of the inflammatory cytokines ([27], p. 16). TNF-polarized macrophages can increase INSL6 peptide expression to promote bone formation in rheumatoid arthritis ([28], p. 2426).

INSL6 methylation in BRCA was evaluated to investigate the association between INSL6 and methylation site, which demonstrated that INSL6 expression was correlated to cg07531356, cg26034799, cg13504907, and cg11830061 in BRCA. Of the different loci tested, Hs_INSL6_03 was identified to contain tissue-specific differential methylation ([29], p. 3079). The specific CG sites adjacent to the CGI of the INSL6 promoter could confer DNA methylation spreading into the CGI, particularly in the setting of KRAB-factor binding ([30], p. 7257). In addition, cisregulatory elements of INSL6 expression facilitate preferential methylation at these promoter CpG islands ([31], p. 2023).

INSL6 expression may be the crosstalk between MI and BRCA due to their high diagnostic and prognostic value as well as the methylation effects on BRCA tumor environments. INSL6 infusion can reduce isoproterenol-induced left ventricular systolic dysfunction and cardiac fibrosis ([4], p. e008441). With each additional CAD risk factor, there was an elevated risk of death (HR, 1.23; 95% CI, 1.08 to 1.40; *P* = 0.002), worse progression-free survival (HR, 1.12; 95% CI, 1.00 to 1.25; *P* = 0.05), and marginally worse cancer-free survival (HR, 1.15; 95% CI, 0.99 to 1.34; *P* = 0.07) for patients with breast cancer ([32], p. 2710), which may be due to the NET formation in atherosclerosis ([33], p. 736) and liver diseases ([7], p. 133) or MI-induced immune response from the bone marrow reservoir ([8], p. 1452). In addition, smoking and breast radiotherapy together were associated with a more than additive effect on the risk of myocardial infarction (HR = 3.04, 95%CI = 2.03 to 4.55; *P* for departure from additivity = 0.039), which in turn exacerbated MI-induced BRCA progression due to treatment toxicity and changes in lifestyle behaviors ([34], p. 365; [35], p. 1435).

In the next step, we will consider the use of the machine learning and deep learning methods in the research of INSL6, such as predicting lncRNA-miRNA-INSL6 or circRNA-miRNA-INSL6 interactions in MI and BRCA progression ([36], p. 874; [37], p. 535; [38], p. bbab286) and using inferring gene regulatory networks to investigate the INSL6 effects ([39], p. 168). Further researches about INSL6 in the crosstalk are still needed. The crosstalk between MI and tumor progression should be investigated, which may be potential biomarkers to impede tumor progression, thus reducing mortality and morbidity.

## 5. Conclusions

INSL6 is differentially expressed after myocardial infarction and also in a variety of tumors and aberrant expression is associated with the progression and immune cell infiltration of the tumor, especially in KIRP and BRCA. Therefore, INSL6 may serve as a potential prognostic biomarker and the crosstalk between MI and tumor progression.

## Figures and Tables

**Table 1:** The significant GO and KEGG pathways enriched by 50 targeting binding proteins.

Ontology	ID	Description	GeneRatio	BgRatio	*P* value	*P*.adjust	*q* value
BP	GO:0060259	Regulation of feeding behavior	3/17	27/18670	1.81*e*-06	7.96*e*-04	6.02*e*-04
BP	GO:0050795	Regulation of behavior	3/17	71/18670	3.45*e*-05	0.005	0.004
BP	GO:2000253	Positive regulation of feeding behavior	2/17	10/18670	3.50*e*-05	0.005	0.004
BP	GO:0007631	Feeding behavior	3/17	101/18670	9.89*e*-05	0.009	0.007
BP	GO:0007218	Neuropeptide signaling pathway	3/17	104/18670	1.08*e*-04	0.009	0.007
MF	GO:0005179	Hormone activity	6/17	122/17697	1.10*e*-09	2.10*e*-08	6.97*e*-09
MF	GO:0048018	Receptor ligand activity	6/17	482/17697	3.79*e*-06	3.60*e*-05	1.20*e*-05
MF	GO:0008528	G protein-coupled peptide receptor activity	4/17	146/17697	9.73*e*-06	5.42*e*-05	1.80*e*-05
MF	GO:0001653	Peptide receptor activity	4/17	152/17697	1.14*e*-05	5.42*e*-05	1.80*e*-05
MF	GO:0004966	Galanin receptor activity	2/17	10/17697	3.89*e*-05	1.48*e*-04	4.91*e*-05
KEGG	hsa04926	Relaxin signaling pathway	7/10	129/8076	2.60*e*-11	8.58*e*-10	7.93*e*-10
KEGG	hsa04080	Neuroactive ligand-receptor interaction	8/10	341/8076	3.90*e*-10	6.43*e*-09	5.95*e*-09

GO: Gene Ontology; BP: biological process; CC: cellular component; MF: molecular function; KEGG: Kyoto Encyclopedia of Genes and Genomes.

**Table 2:** The significant GO and KEGG pathways enriched by top 50 coexpression genes positively related to INSL6 in BRCA.

Ontology	ID	Description	GeneRatio	BgRatio	*P* value	*P*.adjust	*q* value
BP	GO:0032467	Positive regulation of cytokinesis	1/2	40/18670	0.004	0.017	0.001
BP	GO:0001580	Detection of chemical stimulus involved in sensory perception of bitter taste	1/2	41/18670	0.004	0.017	0.001
BP	GO:0050913	Sensory perception of bitter taste	1/2	45/18670	0.005	0.017	0.001
BP	GO:0050912	Detection of chemical stimulus involved in sensory perception of taste	1/2	47/18670	0.005	0.017	0.001
BP	GO:0050909	Sensory perception of taste	1/2	67/18670	0.007	0.017	0.001
MF	GO:0033038	Bitter taste receptor activity	1/3	23/17697	0.004	0.025	0.021
MF	GO:0008527	Taste receptor activity	1/3	29/17697	0.005	0.025	0.021
MF	GO:0003924	GTPase activity	1/3	324/17697	0.054	0.065	0.055
MF	GO:0005525	GTP binding	1/3	374/17697	0.062	0.065	0.055
MF	GO:0032550	Purine ribonucleoside binding	1/3	378/17697	0.063	0.065	0.055
KEGG	hsa04742	Taste transduction	1/1	86/8076	0.011	0.011	

GO: Gene Ontology; BP: biological process; CC: cellular component; MF: molecular function; KEGG: Kyoto Encyclopedia of Genes and Genomes.

**Table 3:** The significant GO and KEGG pathways enriched by top 50 coexpression genes negatively related to INSL6 in BRCA.

Ontology	ID	Description	GeneRatio	BgRatio	*P* value	*P*.adjust	*q* value
CC	GO:0030176	Integral component of endoplasmic reticulum membrane	4/44	150/19717	3.45*e*-04	0.034	0.027
CC	GO:0031227	Intrinsic component of endoplasmic reticulum membrane	4/44	158/19717	4.20*e*-04	0.034	0.027
CC	GO:0033177	Proton-transporting two-sector ATPase complex, proton-transporting domain	2/44	23/19717	0.001	0.064	0.051
MF	GO:0019211	Phosphatase activator activity	2/44	16/17697	7.09*e*-04	0.055	0.046
MF	GO:0046933	Proton-transporting ATP synthase activity, rotational mechanism	2/44	18/17697	9.01*e*-04	0.055	0.046

GO: Gene Ontology; BP: biological process; CC: cellular component; MF: molecular function; KEGG: Kyoto Encyclopedia of Genes and Genomes.

**Table 4:** The significant GO and KEGG pathways enriched by DEGs between INSL6 high- and low-expression groups in BRCA.

Ontology	ID	Description	GeneRatio	BgRatio	*P* value	*P*.adjust	*q* value
BP	GO:0000395	mRNA 5′-splice site recognition	11/371	29/18670	4.13*e*-12	1.01*e*-08	1.00*e*-08
BP	GO:0006376	mRNA splice site selection	12/371	53/18670	4.07*e*-10	4.12*e*-07	4.08*e*-07
BP	GO:0000353	Formation of quadruple SL/U4/U5/U6 snRNP	7/371	12/18670	8.41*e*-10	4.12*e*-07	4.08*e*-07
BP	GO:0000365	mRNA transsplicing, via spliceosome	7/371	12/18670	8.41*e*-10	4.12*e*-07	4.08*e*-07
BP	GO:0045291	mRNA transsplicing, SL addition	7/371	12/18670	8.41*e*-10	4.12*e*-07	4.08*e*-07
CC	GO:0097525	Spliceosomal snRNP complex	18/398	99/19717	1.19*e*-12	3.29*e*-10	2.95*e*-10
CC	GO:0030532	Small nuclear ribonucleoprotein complex	18/398	105/19717	3.40*e*-12	4.70*e*-10	4.21*e*-10
CC	GO:0120114	Sm-like protein family complex	18/398	116/19717	1.95*e*-11	1.79*e*-09	1.61*e*-09
CC	GO:0000786	Nucleosome	17/398	107/19717	4.83*e*-11	3.33*e*-09	2.99*e*-09
CC	GO:0044815	DNA packaging complex	17/398	115/19717	1.57*e*-10	8.65*e*-09	7.75*e*-09
MF	GO:0030627	Pre-mRNA 5′-splice site binding	11/252	24/17697	8.32*e*-15	3.02*e*-12	2.68*e*-12
MF	GO:0033038	Bitter taste receptor activity	9/252	23/17697	1.43*e*-11	2.60*e*-09	2.31*e*-09
MF	GO:0036002	Pre-mRNA binding	12/252	63/17697	7.50*e*-11	9.08*e*-09	8.06*e*-09
MF	GO:0008527	Taste receptor activity	9/252	29/17697	1.63*e*-10	1.48*e*-08	1.31*e*-08
MF	GO:0017075	Syntaxin-1 binding	6/252	26/17697	1.42*e*-06	1.03*e*-04	9.17*e*-05
KEGG	hsa04742	Taste transduction	11/125	86/8076	7.19*e*-08	9.70*e*-06	8.93*e*-06
KEGG	hsa05322	Systemic lupus erythematosus	13/125	136/8076	1.51*e*-07	1.02*e*-05	9.38*e*-06
KEGG	hsa05034	Alcoholism	13/125	187/8076	5.75*e*-06	2.59*e*-04	2.38*e*-04
KEGG	hsa04740	Olfactory transduction	18/125	447/8076	1.65*e*-04	0.006	0.005
KEGG	hsa03013	RNA transport	9/125	186/8076	0.002	0.062	0.057

GO: Gene Ontology; BP: biological process; CC: cellular component; MF: molecular function; KEGG: Kyoto Encyclopedia of Genes and Genomes.

**Table 5:** The gene set enrichment analysis of DEGs between INSL6 high- and low-expression groups in BRCA.

ID	Set Size	Enrichment score	NES	*P* value	*P*.adjust	*q* values	Rank
REACTOME_INNATE_IMMUNE_SYSTEM	10	-0.683638117	-2.68486435	0.005050505	0.184065934	0.170618855	78
REACTOME_NEURONAL_SYSTEM	11	-0.735714911	-3.078729754	0.005494505	0.184065934	0.170618855	155
REACTOME_G_ALPHA_S_SIGNALLING_EVENTS	22	-0.338353414	-1.995658479	0.010989011	0.245421245	0.227491806	1341
REACTOME_OLFACTORY_SIGNALING_PATHWAY	17	-0.337506259	-1.796016849	0.016666667	0.279166667	0.25877193	1341

## Data Availability

Previously reported GEO data were used to support this study and are available in GSE24519 and the BRCA, and other cancer data were all from TCGA.

## Abbreviations

INSL6: Insulin-like peptide 6
CAD: Coronary artery disease
MI: Myocardial infarction
FAMI: First acute myocardial infarction
STEMI: ST-segment elevation myocardial infarction
NET: Neutrophil extracellular trap
HF: Heart failure
GEO: Gene Expression Omnibus
DEGs: Differentially expressed genes
GO: Gene Ontology
KEGG: Kyoto Encyclopedia of Genes and Genomes
GSEA: Gene set enrichment analysis
PPI: Protein-protein interaction
ROC: Receiver operation characteristic
AUC: Area under the curve
ACC: Adrenocortical carcinoma
COAD: Colon adenocarcinoma
DLBC: Lymphoid neoplasm diffuse large B-cell lymphoma
ESCA: Esophageal carcinoma
GBM: Glioblastoma multiforme
KIRP: Kidney renal papillary cell carcinoma
LAML: Acute myeloid leukemia
LUAD: Lung adenocarcinoma
LUSC: Lung squamous cell carcinoma
OV: Ovarian serous cystadenocarcinoma
PCPG: Pheochromocytoma and paraganglioma
READ: Rectum adenocarcinoma
SKCM: Skin cutaneous melanoma
STAD: Stomach adenocarcinoma
TGCT: Testicular germ cell tumors
BRCA: Breast invasive carcinoma
KICH: Kidney chromophobe
KIRC: Kidney renal clear cell carcinoma
LIHC: Liver hepatocellular carcinoma
THCA: Thyroid carcinoma
HR: Hazard ratio
IDC: Infiltrating ductal carcinoma.

## Supplementary Materials

Supplementary Materials Supplementary Table Legend Supplementary Table 1: the same transcripts among DEGs of FAMI A, FAMI B, and TCGA-BRCA. Supplementary Table 2: clinical characteristics of BRCA patients. Supplementary Figure Legend Supplementary Figure 1: the overall survival analysis of other screened genes in BRCA, including ODAM (A), TH (B), IRS4 (C), HOXD13 (D), KLK7 (E), AMER3 (F), GJB6 (G), and FOXG1 (H). Supplementary Figure 2: the diagnostic value of INSL6 in LUAD (A), THCA (B), ACC (C), SKCM (D), GBM (E), DLBC (F), and READ (G). Supplementary Figure 3: the prognostic value of INSL6 in pancancer. (A–C) The overall survival analysis (A), disease-specific survival analysis (B), and progress-free interval analysis (C) of the INSL6 expression in TGCT. (D–F) The overall survival analysis (D), disease-specific survival analysis (E), and progress-free interval analysis (F) of the INSL6 expression in STAD. (G–I) The overall survival analysis (G), disease-specific survival analysis (H), and progress-free interval analysis (I) of the INSL6 expression in ESCA. (J–L) The overall survival analysis (J), disease-specific survival analysis (K), and progress-free interval analysis (L) of the INSL6 expression in OV. Supplementary Figure 4: the prognostic value of INSL6 in pancancer. (A–C) The overall survival analysis (A), disease-specific survival analysis (B), and progress-free interval analysis (C) of the INSL6 expression in KICH. (D–F) The overall survival analysis (D), disease-specific survival analysis (E), and progress-free interval analysis (F) of the INSL6 expression in KIRC. (G) The overall survival analysis of the INSL6 expression in LAML. Supplementary Figure 5: the association between INSL6 expression and immune cells in KIRP using immune infiltration analysis. Supplementary Figure 6: the association between INSL6 expression and immune cells in BRCA using immune infiltration analysis. Supplementary Figure 7: the associations between INSL6 expression and the disease-specific survival in different clinical subgroups of BRCA. (A) Race: white; (B) ER status: negative; (C) M stage: M0; (D) PAM50: Her2; and (E) N stage: N1.
