# Supplementary material for: Myocardial Infarction-Induced INSL6 Decrease Contributes to Breast Cancer Progression
Source: Dis Markers. 2023 Feb 7;2023:8702914. doi: 10.1155/2023/8702914 (PMC9928516; doi:10.1155/2023/8702914)
Supplement: Supplementary Materials — Supplementary Table Legend Supplementary Table 1: the same transcripts among DEGs of FAMI A, FAMI B, and TCGA-BRCA. Supplementary Table 2: clinical characteristics of BRCA patients. Supplementary Figure Legend Supplementary Figure 1: the overall survival analysis of other screened genes in BRCA, including ODAM (A), TH (B), IRS4 (C), HOXD13 (D), KLK7 (E), AMER3 (F), GJB6 (G), and FOXG1 (H). Supplementary Figure 2: the diagnostic value of INSL6 in LUAD (A), THCA (B), ACC (C), SKCM (D), GBM (E), DLBC (F), and READ (G). Supplementary Figure 3: the prognostic value of INSL6 in pancancer. (A–C) The overall survival analysis (A), disease-specific survival analysis (B), and progress-free interval analysis (C) of the INSL6 expression in TGCT. (D–F) The overall survival analysis (D), disease-specific survival analysis (E), and progress-free interval analysis (F) of the INSL6 expression in STAD. (G–I) The overall survival analysis (G), disease-specific survival analysis (H), and progress-free interval analysis (I) of the INSL6 expression in ESCA. (J–L) The overall survival analysis (J), disease-specific survival analysis (K), and progress-free interval analysis (L) of the INSL6 expression in OV. Supplementary Figure 4: the prognostic value of INSL6 in pancancer. (A–C) The overall survival analysis (A), disease-specific survival analysis (B), and progress-free interval analysis (C) of the INSL6 expression in KICH. (D–F) The overall survival analysis (D), disease-specific survival analysis (E), and progress-free interval analysis (F) of the INSL6 expression in KIRC. (G) The overall survival analysis of the INSL6 expression in LAML. Supplementary Figure 5: the association between INSL6 expression and immune cells in KIRP using immune infiltration analysis. Supplementary Figure 6: the association between INSL6 expression and immune cells in BRCA using immune infiltration analysis. Supplementary Figure 7: the associations between INSL6 expression and the disease-specific survival i [file 8702914.f1.zip › Supplementary Figure 7.pdf]

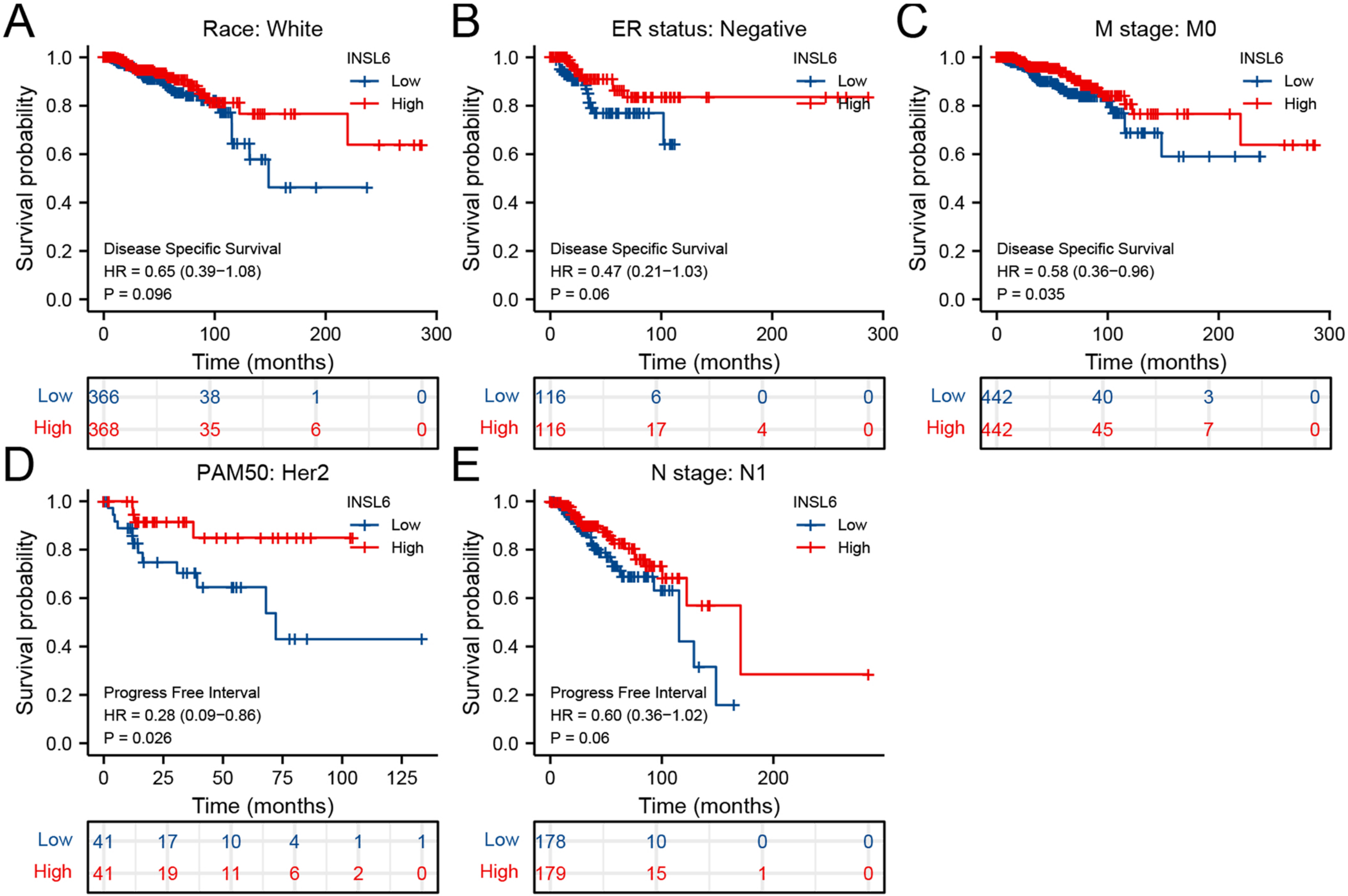

A
Race: White
INSL6
Low
High
Disease Specific Survival
HR = 0.65 (0.39−1.08)
P = 0.096
Survival probability
Time (months)
Low 366 38 1 0
High 368 35 6 0
B
ER status: Negative
INSL6
Low
High
Disease Specific Survival
HR = 0.47 (0.21−1.03)
P = 0.06
Low 116 6 0 0
High 116 17 4 0
C
M stage: M0
INSL6
Low
High
Disease Specific Survival
HR = 0.58 (0.36−0.96)
P = 0.035
Low 442 40 3 0
High 442 45 7 0
D
PAM50: Her2
INSL6
Low
High
Progress Free Interval
HR = 0.28 (0.09−0.86)
P = 0.026
Low 41 17 10 4 1 1
High 41 19 11 6 2 0
E
N stage: N1
INSL6
Low
High
Progress Free Interval
HR = 0.60 (0.36−1.02)
P = 0.06
Low 178 10 0 0
High 179 15 1 0
