# Supplementary material for: Myocardial Infarction-Induced INSL6 Decrease Contributes to Breast Cancer Progression
Source: Dis Markers. 2023 Feb 7;2023:8702914. doi: 10.1155/2023/8702914 (PMC9928516; doi:10.1155/2023/8702914)
Supplement: Supplementary Materials — Supplementary Table Legend Supplementary Table 1: the same transcripts among DEGs of FAMI A, FAMI B, and TCGA-BRCA. Supplementary Table 2: clinical characteristics of BRCA patients. Supplementary Figure Legend Supplementary Figure 1: the overall survival analysis of other screened genes in BRCA, including ODAM (A), TH (B), IRS4 (C), HOXD13 (D), KLK7 (E), AMER3 (F), GJB6 (G), and FOXG1 (H). Supplementary Figure 2: the diagnostic value of INSL6 in LUAD (A), THCA (B), ACC (C), SKCM (D), GBM (E), DLBC (F), and READ (G). Supplementary Figure 3: the prognostic value of INSL6 in pancancer. (A–C) The overall survival analysis (A), disease-specific survival analysis (B), and progress-free interval analysis (C) of the INSL6 expression in TGCT. (D–F) The overall survival analysis (D), disease-specific survival analysis (E), and progress-free interval analysis (F) of the INSL6 expression in STAD. (G–I) The overall survival analysis (G), disease-specific survival analysis (H), and progress-free interval analysis (I) of the INSL6 expression in ESCA. (J–L) The overall survival analysis (J), disease-specific survival analysis (K), and progress-free interval analysis (L) of the INSL6 expression in OV. Supplementary Figure 4: the prognostic value of INSL6 in pancancer. (A–C) The overall survival analysis (A), disease-specific survival analysis (B), and progress-free interval analysis (C) of the INSL6 expression in KICH. (D–F) The overall survival analysis (D), disease-specific survival analysis (E), and progress-free interval analysis (F) of the INSL6 expression in KIRC. (G) The overall survival analysis of the INSL6 expression in LAML. Supplementary Figure 5: the association between INSL6 expression and immune cells in KIRP using immune infiltration analysis. Supplementary Figure 6: the association between INSL6 expression and immune cells in BRCA using immune infiltration analysis. Supplementary Figure 7: the associations between INSL6 expression and the disease-specific survival i [file 8702914.f1.zip › Supplementary Table.docx]

Supplementary Table 1. The same transcripts among DEGs of FAMI A, FAMI B and TCGA-BRCA.

| FAMI A AND BRCA NOT FAMI B | FAMI B AND BRCA NOT FAMI A | FAMI A AND FAMI B AND BRCA |
| --- | --- | --- |
| \| KLK7 \| \| --- \| \| DCAF4L2 \| \| HOXD13 \| \| TH \| | \| CARD18 \| \| --- \| \| IRS4 \| | \| LALBA \| \| --- \| \| CRNN \| \| SSX3 \| \| FOXG1 \| \| INSL6 \| \| BPIFA1 \| \| GJB6 \| \| AMER3 \| |

Supplementary Table 2. Clinical characteristics of BRCA patients.

| Characteristic | Low expression of INSL6 | High expression of INSL6 | p |
| --- | --- | --- | --- |
| n | 541 | 542 |  |
| T stage, n (%) |  |  | 0.217 |
| T1 | 130 (12%) | 147 (13.6%) |  |
| T2 | 330 (30.6%) | 299 (27.7%) |  |
| T3 | 62 (5.7%) | 77 (7.1%) |  |
| T4 | 19 (1.8%) | 16 (1.5%) |  |
| N stage, n (%) |  |  | 0.973 |
| N0 | 256 (24.1%) | 258 (24.2%) |  |
| N1 | 181 (17%) | 177 (16.6%) |  |
| N2 | 56 (5.3%) | 60 (5.6%) |  |
| N3 | 37 (3.5%) | 39 (3.7%) |  |
| M stage, n (%) |  |  | 0.890 |
| M0 | 459 (49.8%) | 443 (48%) |  |
| M1 | 11 (1.2%) | 9 (1%) |  |
| Pathologic stage, n (%) |  |  | 0.939 |
| Stage I | 89 (8.4%) | 92 (8.7%) |  |
| Stage II | 309 (29.2%) | 310 (29.2%) |  |
| Stage III | 124 (11.7%) | 118 (11.1%) |  |
| Stage IV | 10 (0.9%) | 8 (0.8%) |  |
| Race, n (%) |  |  | 0.296 |
| Asian | 36 (3.6%) | 24 (2.4%) |  |
| Black or African American | 91 (9.2%) | 90 (9.1%) |  |
| White | 373 (37.5%) | 380 (38.2%) |  |
| Age, meidan (IQR) | 59 (50, 68) | 57 (47, 66) | 0.033 |
| Age, n (%) |  |  | 0.286 |
| <=60 | 291 (26.9%) | 310 (28.6%) |  |
| >60 | 250 (23.1%) | 232 (21.4%) |  |
| Histological type, n (%) |  |  | 0.005 |
| Infiltrating Ductal Carcinoma | 404 (41.4%) | 368 (37.7%) |  |
| Infiltrating Lobular Carcinoma | 84 (8.6%) | 121 (12.4%) |  |
| PR status, n (%) |  |  | 0.785 |
| Negative | 176 (17%) | 166 (16.1%) |  |
| Indeterminate | 2 (0.2%) | 2 (0.2%) |  |
| Positive | 335 (32.4%) | 353 (34.1%) |  |
| ER status, n (%) |  |  | 0.370 |
| Negative | 123 (11.9%) | 117 (11.3%) |  |
| Indeterminate | 2 (0.2%) | 0 (0%) |  |
| Positive | 389 (37.6%) | 404 (39%) |  |
| HER2 status, n (%) |  |  | 0.125 |
| Negative | 272 (37.4%) | 286 (39.3%) |  |
| Indeterminate | 6 (0.8%) | 6 (0.8%) |  |
| Positive | 91 (12.5%) | 66 (9.1%) |  |
| PAM50, n (%) |  |  | < 0.001 |
| Normal | 16 (1.5%) | 24 (2.2%) |  |
| LumA | 252 (23.3%) | 310 (28.6%) |  |
| LumB | 122 (11.3%) | 82 (7.6%) |  |
| Her2 | 52 (4.8%) | 30 (2.8%) |  |
| Basal | 99 (9.1%) | 96 (8.9%) |  |
| Menopause status, n (%) |  |  | 0.020 |
| Pre | 96 (9.9%) | 133 (13.7%) |  |
| Peri | 21 (2.2%) | 19 (2%) |  |
| Post | 369 (38%) | 334 (34.4%) |  |
| Anatomic neoplasm subdivisions, n (%) |  |  | 0.121 |
| Left | 268 (24.7%) | 295 (27.2%) |  |
| Right | 273 (25.2%) | 247 (22.8%) |  |
| radiation_therapy, n (%) |  |  | 0.137 |
| No | 228 (23.1%) | 206 (20.9%) |  |
| Yes | 263 (26.6%) | 290 (29.4%) |  |
| OS event, n (%) |  |  | 0.043 |
| Alive | 453 (41.8%) | 478 (44.1%) |  |
| Dead | 88 (8.1%) | 64 (5.9%) |  |
| DSS event, n (%) |  |  | 0.166 |
| Alive | 481 (45.2%) | 497 (46.8%) |  |
| Dead | 49 (4.6%) | 36 (3.4%) |  |
| PFI event, n (%) |  |  | 0.850 |
| Alive | 466 (43%) | 470 (43.4%) |  |
| Dead | 75 (6.9%) | 72 (6.6%) |  |
