# Supplementary figures and images for: Myocardial Infarction-Induced INSL6 Decrease Contributes to Breast Cancer Progression
Source: Dis Markers. 2023 Feb 7;2023:8702914. doi: 10.1155/2023/8702914 (PMC9928516; doi:10.1155/2023/8702914)

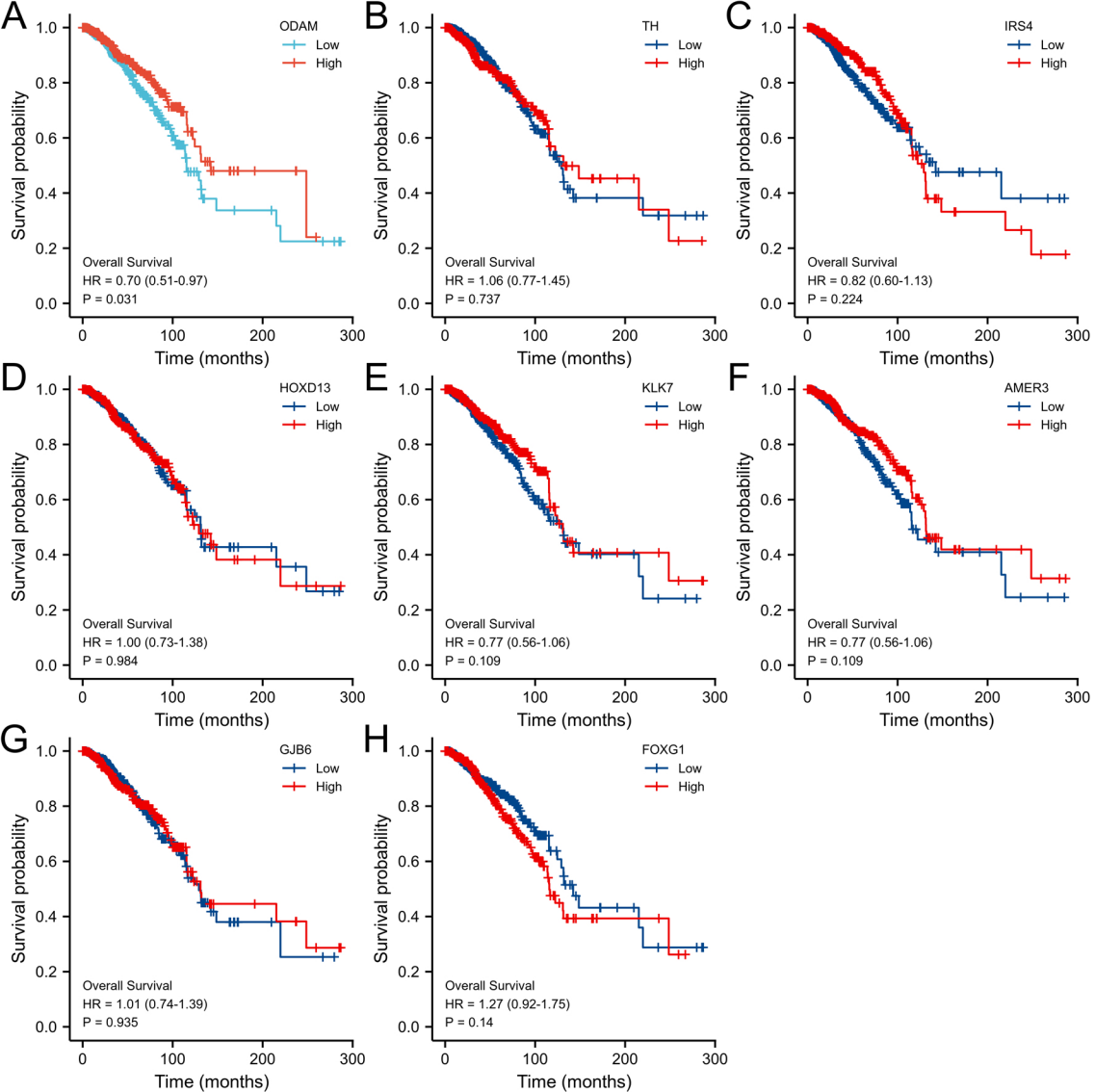

Supplement: Supplementary Materials — Supplementary Table Legend Supplementary Table 1: the same transcripts among DEGs of FAMI A, FAMI B, and TCGA-BRCA. Supplementary Table 2: clinical characteristics of BRCA patients. Supplementary Figure Legend Supplementary Figure 1: the overall survival analysis of other screened genes in BRCA, including ODAM (A), TH (B), IRS4 (C), HOXD13 (D), KLK7 (E), AMER3 (F), GJB6 (G), and FOXG1 (H). Supplementary Figure 2: the diagnostic value of INSL6 in LUAD (A), THCA (B), ACC (C), SKCM (D), GBM (E), DLBC (F), and READ (G). Supplementary Figure 3: the prognostic value of INSL6 in pancancer. (A–C) The overall survival analysis (A), disease-specific survival analysis (B), and progress-free interval analysis (C) of the INSL6 expression in TGCT. (D–F) The overall survival analysis (D), disease-specific survival analysis (E), and progress-free interval analysis (F) of the INSL6 expression in STAD. (G–I) The overall survival analysis (G), disease-specific survival analysis (H), and progress-free interval analysis (I) of the INSL6 expression in ESCA. (J–L) The overall survival analysis (J), disease-specific survival analysis (K), and progress-free interval analysis (L) of the INSL6 expression in OV. Supplementary Figure 4: the prognostic value of INSL6 in pancancer. (A–C) The overall survival analysis (A), disease-specific survival analysis (B), and progress-free interval analysis (C) of the INSL6 expression in KICH. (D–F) The overall survival analysis (D), disease-specific survival analysis (E), and progress-free interval analysis (F) of the INSL6 expression in KIRC. (G) The overall survival analysis of the INSL6 expression in LAML. Supplementary Figure 5: the association between INSL6 expression and immune cells in KIRP using immune infiltration analysis. Supplementary Figure 6: the association between INSL6 expression and immune cells in BRCA using immune infiltration analysis. Supplementary Figure 7: the associations between INSL6 expression and the disease-specific survival i [file 8702914.f1.zip › Supplementary Figure 1.pdf]

**A**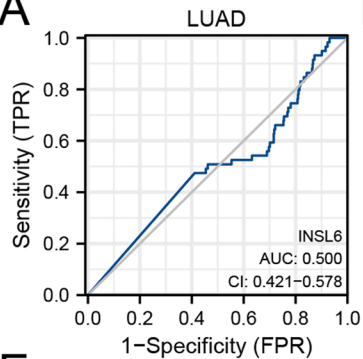**B**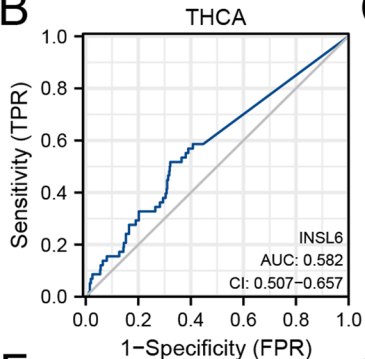**C**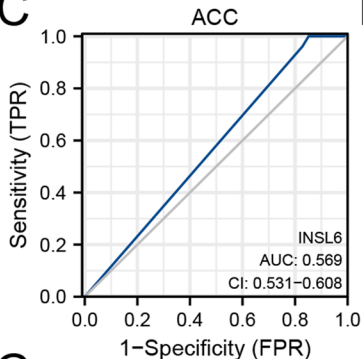**D**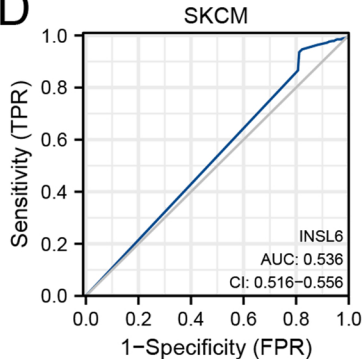**E**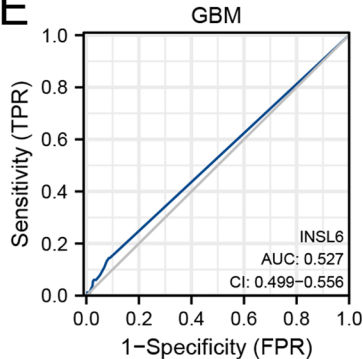**F**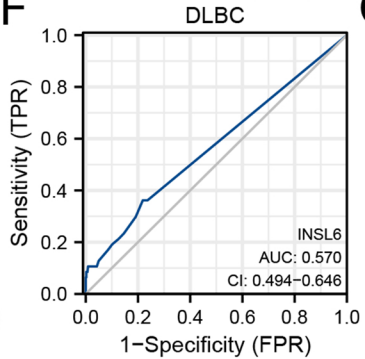**G**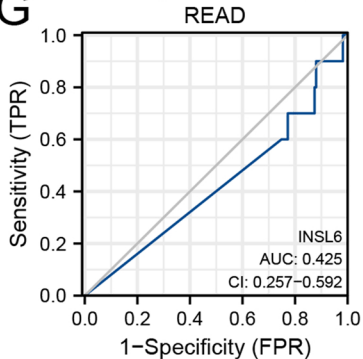

Supplement: Supplementary Materials — Supplementary Table Legend Supplementary Table 1: the same transcripts among DEGs of FAMI A, FAMI B, and TCGA-BRCA. Supplementary Table 2: clinical characteristics of BRCA patients. Supplementary Figure Legend Supplementary Figure 1: the overall survival analysis of other screened genes in BRCA, including ODAM (A), TH (B), IRS4 (C), HOXD13 (D), KLK7 (E), AMER3 (F), GJB6 (G), and FOXG1 (H). Supplementary Figure 2: the diagnostic value of INSL6 in LUAD (A), THCA (B), ACC (C), SKCM (D), GBM (E), DLBC (F), and READ (G). Supplementary Figure 3: the prognostic value of INSL6 in pancancer. (A–C) The overall survival analysis (A), disease-specific survival analysis (B), and progress-free interval analysis (C) of the INSL6 expression in TGCT. (D–F) The overall survival analysis (D), disease-specific survival analysis (E), and progress-free interval analysis (F) of the INSL6 expression in STAD. (G–I) The overall survival analysis (G), disease-specific survival analysis (H), and progress-free interval analysis (I) of the INSL6 expression in ESCA. (J–L) The overall survival analysis (J), disease-specific survival analysis (K), and progress-free interval analysis (L) of the INSL6 expression in OV. Supplementary Figure 4: the prognostic value of INSL6 in pancancer. (A–C) The overall survival analysis (A), disease-specific survival analysis (B), and progress-free interval analysis (C) of the INSL6 expression in KICH. (D–F) The overall survival analysis (D), disease-specific survival analysis (E), and progress-free interval analysis (F) of the INSL6 expression in KIRC. (G) The overall survival analysis of the INSL6 expression in LAML. Supplementary Figure 5: the association between INSL6 expression and immune cells in KIRP using immune infiltration analysis. Supplementary Figure 6: the association between INSL6 expression and immune cells in BRCA using immune infiltration analysis. Supplementary Figure 7: the associations between INSL6 expression and the disease-specific survival i [file 8702914.f1.zip › Supplementary Figure 2.pdf]

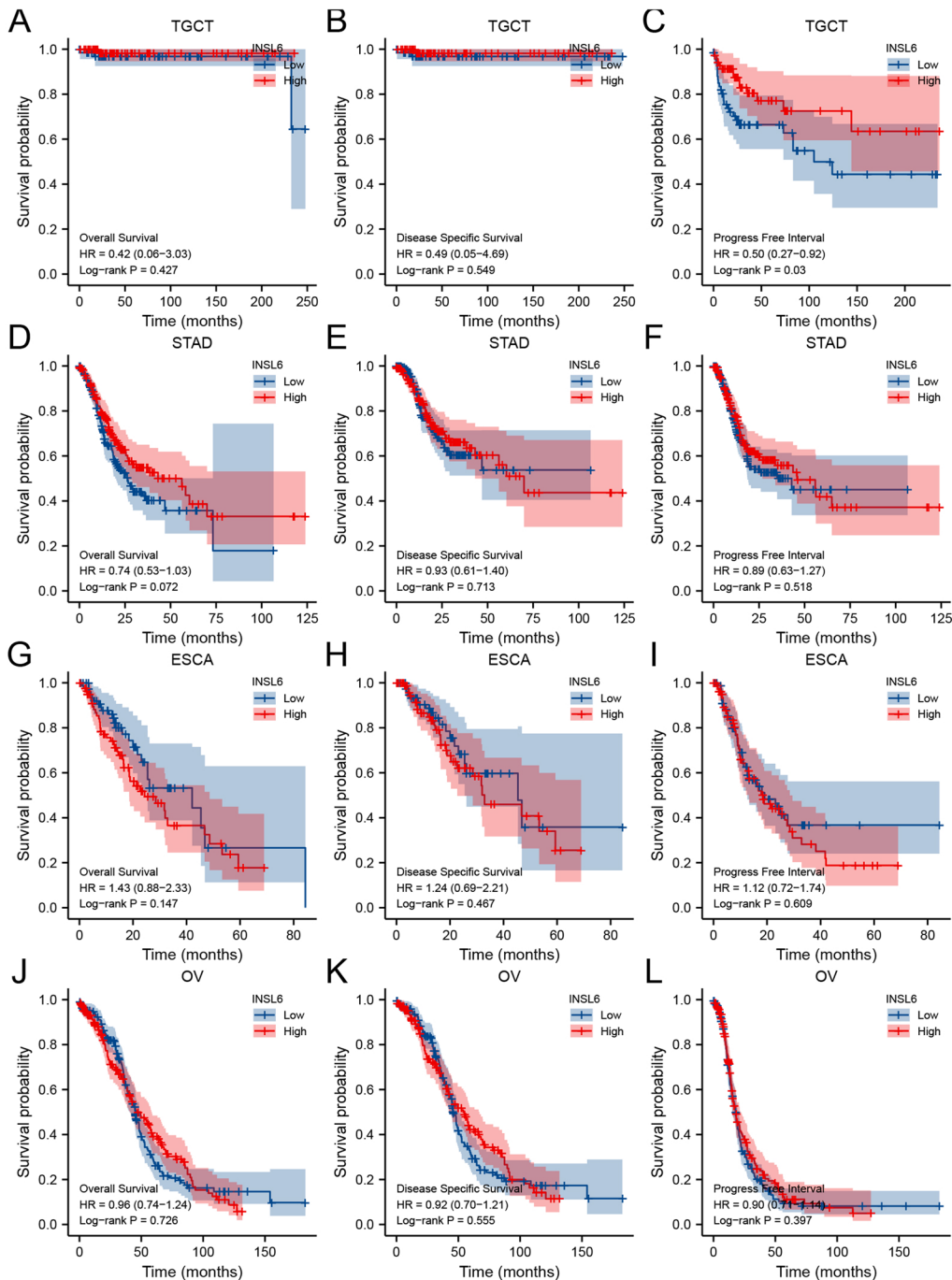

Supplement: Supplementary Materials — Supplementary Table Legend Supplementary Table 1: the same transcripts among DEGs of FAMI A, FAMI B, and TCGA-BRCA. Supplementary Table 2: clinical characteristics of BRCA patients. Supplementary Figure Legend Supplementary Figure 1: the overall survival analysis of other screened genes in BRCA, including ODAM (A), TH (B), IRS4 (C), HOXD13 (D), KLK7 (E), AMER3 (F), GJB6 (G), and FOXG1 (H). Supplementary Figure 2: the diagnostic value of INSL6 in LUAD (A), THCA (B), ACC (C), SKCM (D), GBM (E), DLBC (F), and READ (G). Supplementary Figure 3: the prognostic value of INSL6 in pancancer. (A–C) The overall survival analysis (A), disease-specific survival analysis (B), and progress-free interval analysis (C) of the INSL6 expression in TGCT. (D–F) The overall survival analysis (D), disease-specific survival analysis (E), and progress-free interval analysis (F) of the INSL6 expression in STAD. (G–I) The overall survival analysis (G), disease-specific survival analysis (H), and progress-free interval analysis (I) of the INSL6 expression in ESCA. (J–L) The overall survival analysis (J), disease-specific survival analysis (K), and progress-free interval analysis (L) of the INSL6 expression in OV. Supplementary Figure 4: the prognostic value of INSL6 in pancancer. (A–C) The overall survival analysis (A), disease-specific survival analysis (B), and progress-free interval analysis (C) of the INSL6 expression in KICH. (D–F) The overall survival analysis (D), disease-specific survival analysis (E), and progress-free interval analysis (F) of the INSL6 expression in KIRC. (G) The overall survival analysis of the INSL6 expression in LAML. Supplementary Figure 5: the association between INSL6 expression and immune cells in KIRP using immune infiltration analysis. Supplementary Figure 6: the association between INSL6 expression and immune cells in BRCA using immune infiltration analysis. Supplementary Figure 7: the associations between INSL6 expression and the disease-specific survival i [file 8702914.f1.zip › Supplementary Figure 3.pdf]

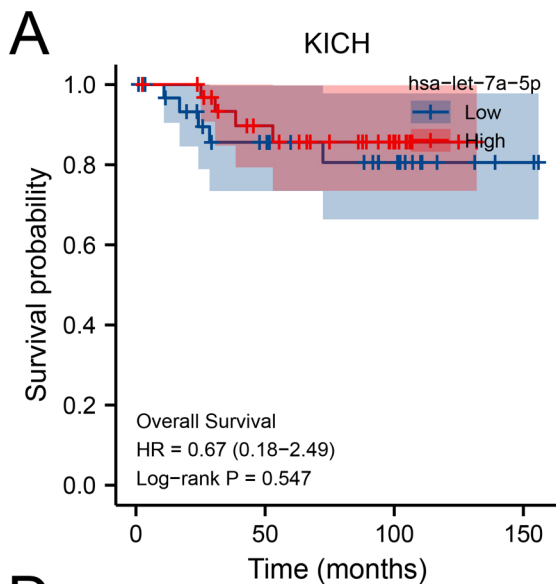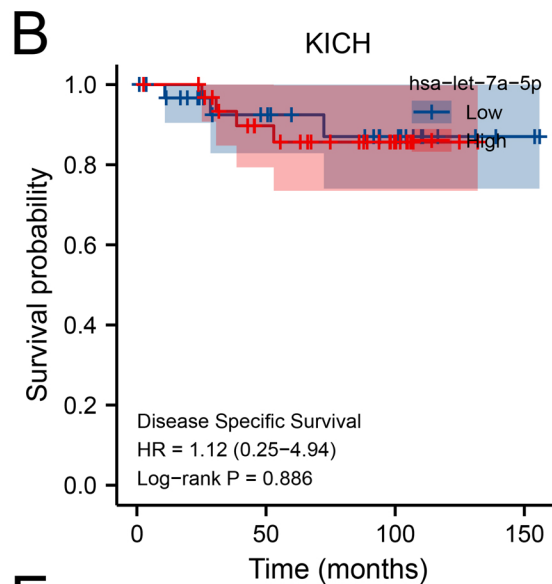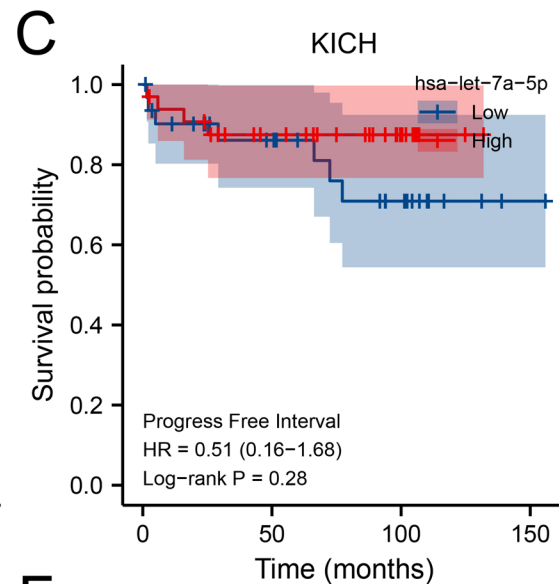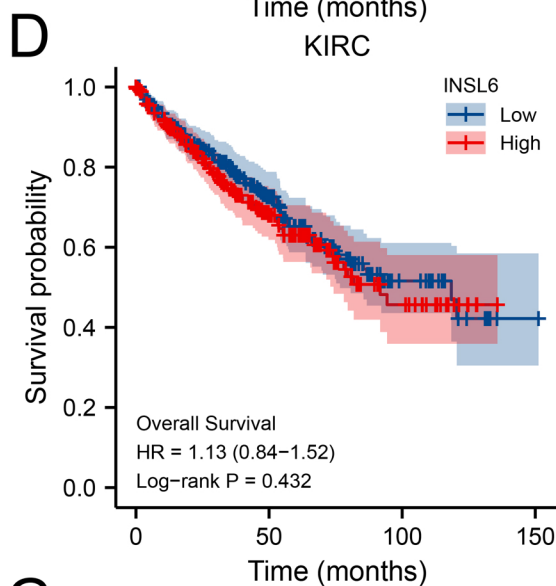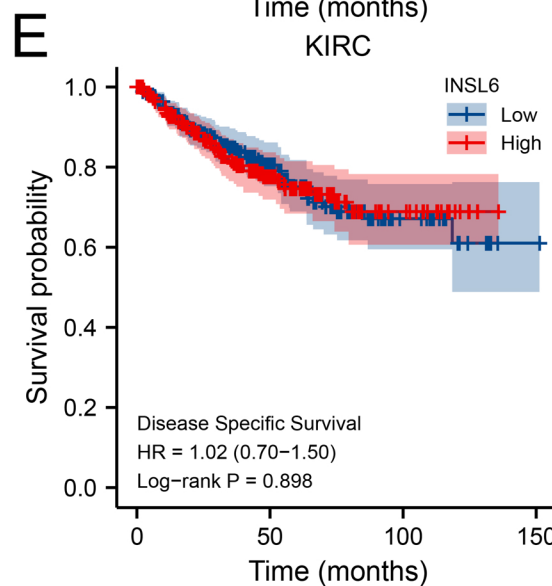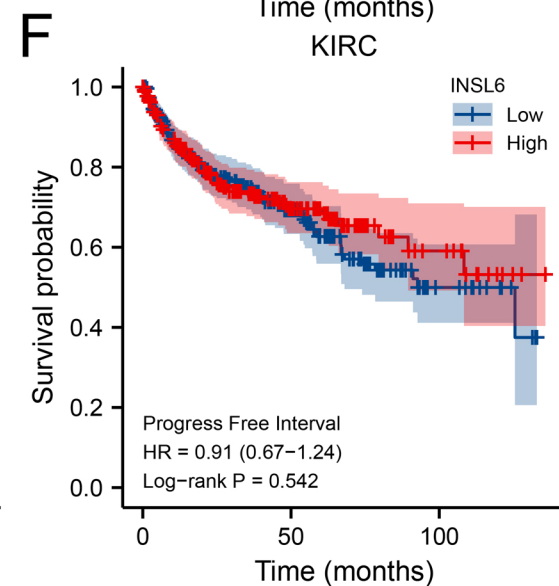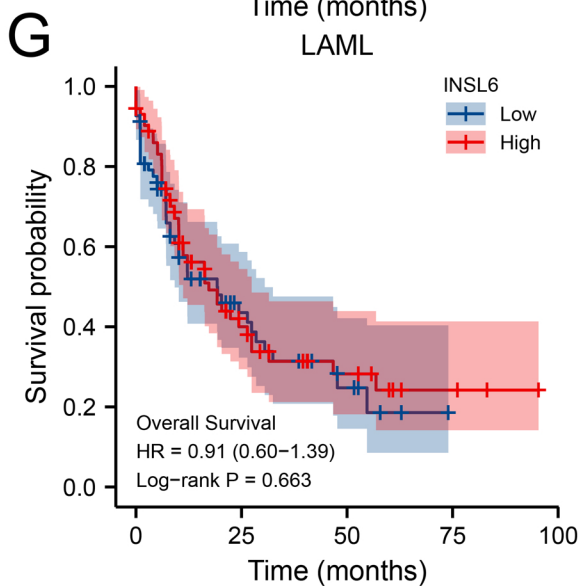

Supplement: Supplementary Materials — Supplementary Table Legend Supplementary Table 1: the same transcripts among DEGs of FAMI A, FAMI B, and TCGA-BRCA. Supplementary Table 2: clinical characteristics of BRCA patients. Supplementary Figure Legend Supplementary Figure 1: the overall survival analysis of other screened genes in BRCA, including ODAM (A), TH (B), IRS4 (C), HOXD13 (D), KLK7 (E), AMER3 (F), GJB6 (G), and FOXG1 (H). Supplementary Figure 2: the diagnostic value of INSL6 in LUAD (A), THCA (B), ACC (C), SKCM (D), GBM (E), DLBC (F), and READ (G). Supplementary Figure 3: the prognostic value of INSL6 in pancancer. (A–C) The overall survival analysis (A), disease-specific survival analysis (B), and progress-free interval analysis (C) of the INSL6 expression in TGCT. (D–F) The overall survival analysis (D), disease-specific survival analysis (E), and progress-free interval analysis (F) of the INSL6 expression in STAD. (G–I) The overall survival analysis (G), disease-specific survival analysis (H), and progress-free interval analysis (I) of the INSL6 expression in ESCA. (J–L) The overall survival analysis (J), disease-specific survival analysis (K), and progress-free interval analysis (L) of the INSL6 expression in OV. Supplementary Figure 4: the prognostic value of INSL6 in pancancer. (A–C) The overall survival analysis (A), disease-specific survival analysis (B), and progress-free interval analysis (C) of the INSL6 expression in KICH. (D–F) The overall survival analysis (D), disease-specific survival analysis (E), and progress-free interval analysis (F) of the INSL6 expression in KIRC. (G) The overall survival analysis of the INSL6 expression in LAML. Supplementary Figure 5: the association between INSL6 expression and immune cells in KIRP using immune infiltration analysis. Supplementary Figure 6: the association between INSL6 expression and immune cells in BRCA using immune infiltration analysis. Supplementary Figure 7: the associations between INSL6 expression and the disease-specific survival i [file 8702914.f1.zip › Supplementary Figure 4.pdf]

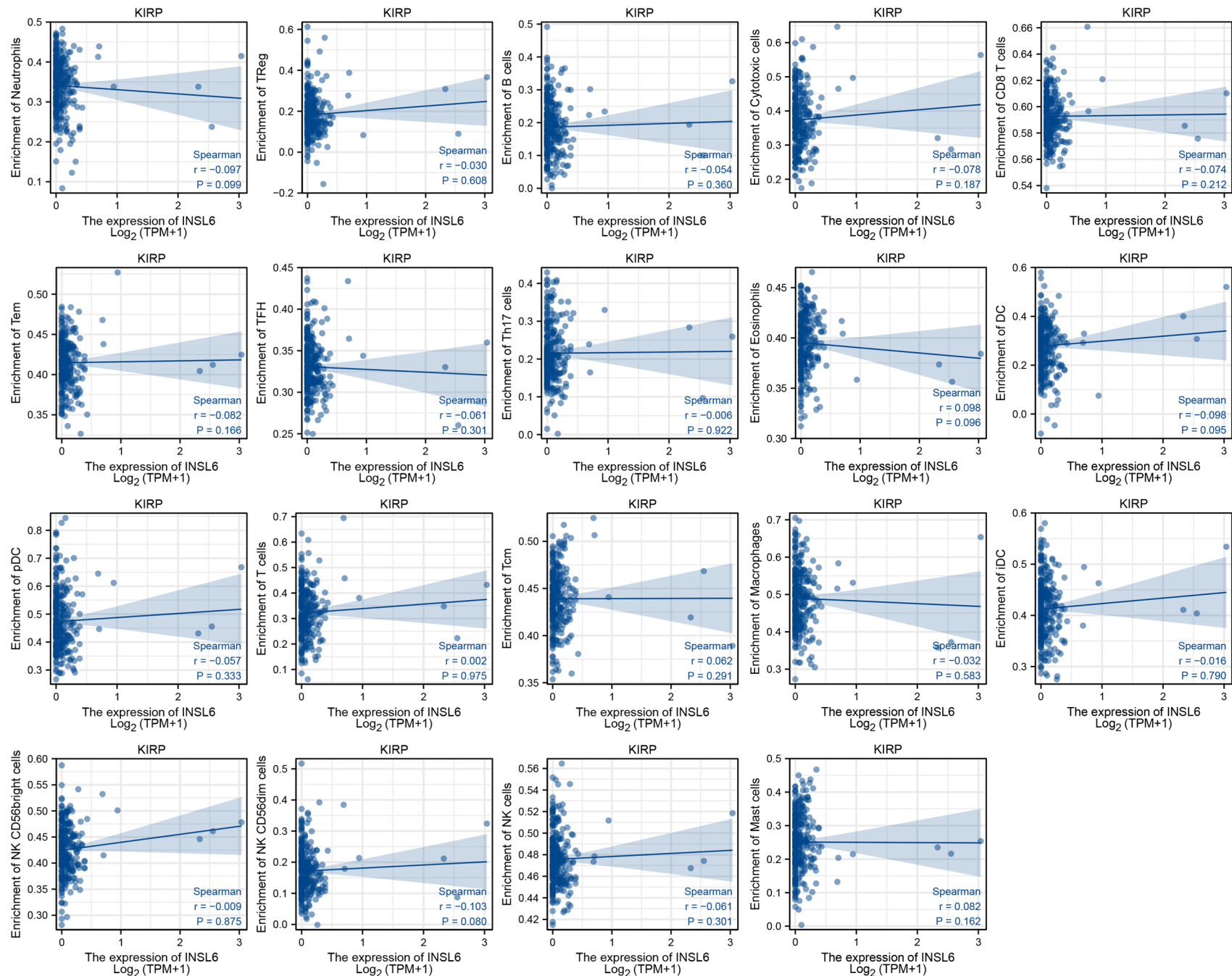

Supplement: Supplementary Materials — Supplementary Table Legend Supplementary Table 1: the same transcripts among DEGs of FAMI A, FAMI B, and TCGA-BRCA. Supplementary Table 2: clinical characteristics of BRCA patients. Supplementary Figure Legend Supplementary Figure 1: the overall survival analysis of other screened genes in BRCA, including ODAM (A), TH (B), IRS4 (C), HOXD13 (D), KLK7 (E), AMER3 (F), GJB6 (G), and FOXG1 (H). Supplementary Figure 2: the diagnostic value of INSL6 in LUAD (A), THCA (B), ACC (C), SKCM (D), GBM (E), DLBC (F), and READ (G). Supplementary Figure 3: the prognostic value of INSL6 in pancancer. (A–C) The overall survival analysis (A), disease-specific survival analysis (B), and progress-free interval analysis (C) of the INSL6 expression in TGCT. (D–F) The overall survival analysis (D), disease-specific survival analysis (E), and progress-free interval analysis (F) of the INSL6 expression in STAD. (G–I) The overall survival analysis (G), disease-specific survival analysis (H), and progress-free interval analysis (I) of the INSL6 expression in ESCA. (J–L) The overall survival analysis (J), disease-specific survival analysis (K), and progress-free interval analysis (L) of the INSL6 expression in OV. Supplementary Figure 4: the prognostic value of INSL6 in pancancer. (A–C) The overall survival analysis (A), disease-specific survival analysis (B), and progress-free interval analysis (C) of the INSL6 expression in KICH. (D–F) The overall survival analysis (D), disease-specific survival analysis (E), and progress-free interval analysis (F) of the INSL6 expression in KIRC. (G) The overall survival analysis of the INSL6 expression in LAML. Supplementary Figure 5: the association between INSL6 expression and immune cells in KIRP using immune infiltration analysis. Supplementary Figure 6: the association between INSL6 expression and immune cells in BRCA using immune infiltration analysis. Supplementary Figure 7: the associations between INSL6 expression and the disease-specific survival i [file 8702914.f1.zip › Supplementary Figure 5.pdf]

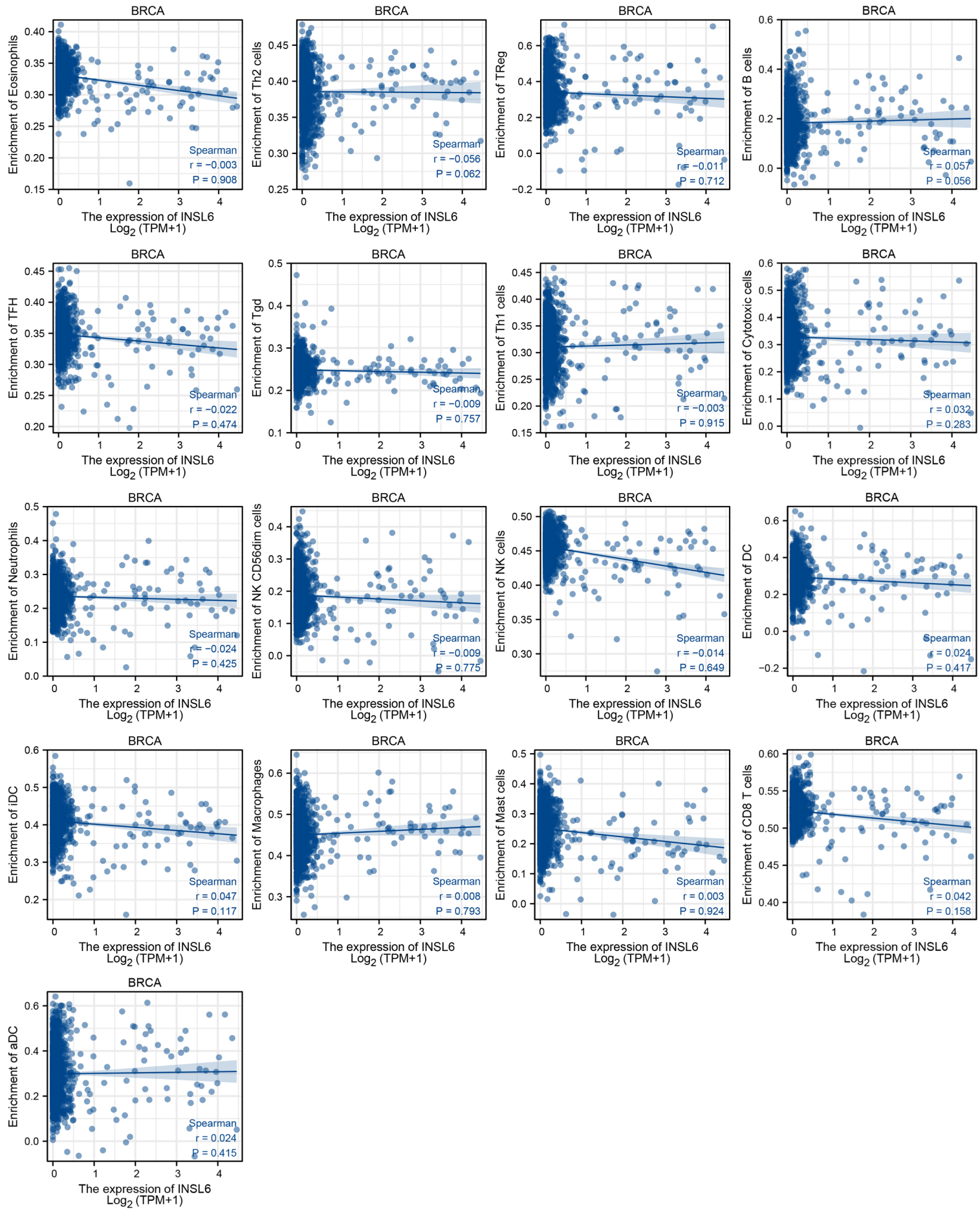

Supplement: Supplementary Materials — Supplementary Table Legend Supplementary Table 1: the same transcripts among DEGs of FAMI A, FAMI B, and TCGA-BRCA. Supplementary Table 2: clinical characteristics of BRCA patients. Supplementary Figure Legend Supplementary Figure 1: the overall survival analysis of other screened genes in BRCA, including ODAM (A), TH (B), IRS4 (C), HOXD13 (D), KLK7 (E), AMER3 (F), GJB6 (G), and FOXG1 (H). Supplementary Figure 2: the diagnostic value of INSL6 in LUAD (A), THCA (B), ACC (C), SKCM (D), GBM (E), DLBC (F), and READ (G). Supplementary Figure 3: the prognostic value of INSL6 in pancancer. (A–C) The overall survival analysis (A), disease-specific survival analysis (B), and progress-free interval analysis (C) of the INSL6 expression in TGCT. (D–F) The overall survival analysis (D), disease-specific survival analysis (E), and progress-free interval analysis (F) of the INSL6 expression in STAD. (G–I) The overall survival analysis (G), disease-specific survival analysis (H), and progress-free interval analysis (I) of the INSL6 expression in ESCA. (J–L) The overall survival analysis (J), disease-specific survival analysis (K), and progress-free interval analysis (L) of the INSL6 expression in OV. Supplementary Figure 4: the prognostic value of INSL6 in pancancer. (A–C) The overall survival analysis (A), disease-specific survival analysis (B), and progress-free interval analysis (C) of the INSL6 expression in KICH. (D–F) The overall survival analysis (D), disease-specific survival analysis (E), and progress-free interval analysis (F) of the INSL6 expression in KIRC. (G) The overall survival analysis of the INSL6 expression in LAML. Supplementary Figure 5: the association between INSL6 expression and immune cells in KIRP using immune infiltration analysis. Supplementary Figure 6: the association between INSL6 expression and immune cells in BRCA using immune infiltration analysis. Supplementary Figure 7: the associations between INSL6 expression and the disease-specific survival i [file 8702914.f1.zip › Supplementary Figure 6.pdf]
